# Anti-Hyperuricemic Effects of Extracts from *Chaenomeles speciosa* (Sweet) Nakai Fruits on Hyperuricemic Rats

**DOI:** 10.3390/metabo14020117

**Published:** 2024-02-10

**Authors:** Ruoling Xu, Peng Deng, Yiren Ma, Kui Li, Fucai Ren, Ning Li

**Affiliations:** Anhui Key Laboratory of Bioactivity of Natural Products, School of Pharmacy, Anhui Medical University, Hefei 230032, China; xuruoling0126@gmail.com (R.X.); deng20232024@gmail.com (P.D.); myr445926585@gmail.com (Y.M.); lkui0169@gmail.com (K.L.)

**Keywords:** *C. speciosa* fruits total extract (CSFTE), hyperuricemia, uric acid, xanthine oxidase

## Abstract

*Chaenomeles speciosa* (Sweet) Nakai (*C. speciosa*) fruit has medicinal and food applications and exhibits beneficial pharmacological properties. This study aimed to explore the hypouricemic effect of *C. speciosa* fruit extracts on hyperuricemic rats and uncover potential protective mechanisms. The rats were given hypoxanthine (HX, 100 mg/kg) and potassium oxonate (PO, 300 mg/kg) for 14 days to induce hyperuricemia. Subsequently, the rats were orally administered *C. speciosa* fruits total extract (CSFTE, 250, 500, and 1000 mg/kg) and allopurinol (AP, 10 mg/kg) one hour after exposure to HX and PO. The results showed that CSFTE had significant xanthine oxidase (XOD) inhibitory activity in vitro (IC_50_ value of 334.2 μg/mL) and exhibited hypouricemic effects in vivo, reducing uric acid (UA), creatinine (CRE), and blood urea nitrogen (BUN) levels in serum. CSFTE increased UA excretion through the regulation of URAT1, GLUT9, OAT1, and OAT3 protein expression in the kidneys of hyperuricemic rats. Additionally, CSFTE (500 and 1000 mg/kg) was more effective than AP in improving renal injury and protecting kidney function in hyperuricemic rats. Our study demonstrated that CSFTE effectively reduced UA levels and protected the kidneys by inhibiting XOD expression in vitro and regulating UA, CRE, BUN, URAT1, GLUT9, OAT1, and OAT3 proteins in vivo.

## 1. Introduction

HUA is a persistent metabolic condition characterized by an irregularity in the metabolism of purine, resulting in elevated levels of uric acid within the bloodstream [1]. The presence of HUA is widely recognized as a major contributing factor to the emergence of gout, hypertension, and diabetes [2]. Due to changes in lifestyle and dietary patterns, the global prevalence of HUA is steadily increasing each year, reaching as high as 17.4% in China [3,4]. The prevalence of HUA was approximately 20% in America and even higher in Korea, up to 26.6% [5]. Consequently, there has been a growing number of studies focusing on HUA.

The main approach to prevent and treat hyperuricemia is to reduce the levels of uric acid (UA) in the circulatory system, which can be accomplished by inhibiting the production of urate or enhancing its elimination. The production of UA is the ultimate outcome of purine metabolism. The conversion of purines into UA occurs in the liver through the action of xanthine oxidase (XOD), which has a significant impact on the production of UA by acting as a key enzyme in its synthesis [6]. Therefore, XOD represents a crucial factor in decreasing the production of UA and its widespread occurrence. The drug used in clinical practice for treating HUA, allopurinol (AP), exerts its therapeutic effect by targeting XOD to lower UA levels [7]. Nonetheless, the potential side effects of AP hinder its clinical applications, notably adverse effects on the kidneys and digestive system, harm to the liver and kidneys, as well as suppression of bone marrow function [8].

The metabolism of urate is heavily influenced by the kidney, which performs four key processes involved in renal function including glomerular filtration, reabsorption within the tubules, secretion, and subsequent reabsorption after secretion. There is growing evidence supporting the claim that proteins linked to urate transporters play a role in the regulation of both urate reabsorption and secretion. The facilitation of urate reabsorption is primarily attributed to the function of the urate transporter 1 (URAT1) and glucose transporter 9 (GLUT9) in cellular transport activities [9]. The transport and metabolism of (UA) are primarily mediated by the pivotal roles played by transporters for organic anions, namely, OAT1 and OAT3 [10,11,12,13].

The fruits of *Chaenomeles sinensis* (Thouin) Koehne (*C. sinensis*) contain a high amount of dietary fiber, natural compounds found in plants such as organic acids, flavonoids, tannins, and bioactive triterpenes like oleanolic acid and ursolic acid. Studies have shown that the extract of *C. sinensis* possesses XOD-inhibitory activity, leading to hypouricemic effects and a reduction in serum UA levels in hyperuricemic mice. These findings indicate that *C. sinensis* could potentially offer advantages in the prevention of hyperuricemia (HUA) and gout [14]. The fruit of *Chaenomeles speciosa* (Sweet) Nakai (*C. speciosa*), commonly referred to as Mugua in China, belongs to the Rosaceae family and falls under the genus *Chaenomeles* [15,16]. *C. speciosa* shares the same family and genus with *C. sinensis*, exhibiting similar main components [17]. Therefore, it is reasonable to believe that the fruits of *C. speciosa* may also exhibit anti-hyperuricemic effects. And the authentic medicinal product recorded in the Chinese pharmacopoeia, *C. speciosa*, is widely circulated in folk medicine and possesses a broad spectrum of therapeutic applications for various diseases [18]. Pharmacological investigations have revealed that the plant exhibits a diverse array of attributes, encompassing anti-inflammatory, pain-relieving, antimicrobial, antioxidant, and immune-regulating actions, neuroprotective effects against Parkinson’s disease, and liver-protective and anticancer properties [19,20,21,22,23]. However, there is currently a dearth of studies and reports investigating the effects of *C. speciosa* in terms of UA reduction and HUA prevention.

Based on the aforementioned findings, *C. speciosa* exhibits potential as a therapeutic agent for managing HUA and warrants further investigation. In this study, we initially conducted an in vitro enzymatic assay to screen for the most effective site of *C. speciosa* extracts that could inhibit XOD activity. Subsequently, an in vivo rat model of HUA was established to investigate the impact of HUA and its potential mechanism.

## 2. Materials and Methods

### 2.1. Materials

Xanthine (X7375), XOD (X4500), and AP (A8003) were supplied by a company based in St. Louis, MO, USA, known as Sigma-Aldrich LLC. The model establishment involved the acquisition of potassium oxonate (PO, 98%, P831461) and hypoxanthine (HX, 99%, H811076) from Macklin Biochemical Co., Ltd. (Shanghai, China). Nanjing Jiancheng Biotechnology Institute (located in Nanjing, China) supplied assay kits used for the determination of UA, creatinine (CRE), and blood urea nitrogen (BUN). Protein antibodies URAT1 (14937-1-AP) and GLUT9 (67530-1-Ig) were obtained from Proteintech Group, Inc. (located in Wuhan, China). Protein antibodies OAT1 (PA5-80017) and OAT3 (PA5-102699) were acquired from Thermo Fisher Scientific-CN (Wuhan, China). And anti-GAPDH (AF7021) was purchased from Affinity Biosciences LTD (Liyang, China). Secondary HRP-labeled goat antibody against rabbit IgG and secondary goat antibody labeled with HRP-targeting rabbit IgG were procured from Yazyme Biotechnology Co., Ltd. (Shanghai, China).

The fruits of *C. speciosa* were provided by Anhui Xiehecheng Chinese Herb Limited Corporration, located in Bozhou, China, in September 2021. The species was first discovered in Sichuan Province, specifically Guangyuan County within Mianyang City, and its identification can be credited to the esteemed Prof. Kai-Jin Wang from Anhui University who submitted a voucher specimen (20210930) to the Pharmacy Department at Anhui Medical University.

### 2.2. Preparation of C. speciosa Fruits Extracts

The plant materials (10 kg) were successively soaked in ethanol solutions (95%, 85%, and 75%, at a ratio of 1:10 *w*/*v*) at ambient temperature for a duration of 12 h, followed by ultrasonic extraction at 60 °C for 3 h at a frequency of 100 Hz for 10 min per hour (Kunshan Jielimei Ultrasonic Instrument Co., Ltd. located in Suzhou, China, KS-600E). Subsequently, the filtrate was mixed and filtered twice with three layers of medical gauze (Nanjing Pharmacy located in Nanjing, China, 40 mesh), and then concentrated with a rotary evaporator (temperature 60 °C, speed 45 rpm) to obtain about 1.8 L of extract. The resulting extract was designated as CSF-E. The filtrate residue obtained after ethanol extraction was subjected to two additional extractions at 80 °C for 3 h using 20 L of distilled water under ultrasound-assisted conditions, followed by filtration. The resulting filtrate was concentrated to obtain about 0.8 L of extract (referred to as CSF-W). Half of the CSF-E and CSF-W were combined, thoroughly mixed, and designated as *C. speciosa* fruits total extract, referred to as CSFTE (1.3 L). Finally, the three extracts were freeze-dried into freeze-dried powder, and 1396 g CSF-E, 923 g CSF-W, and 2311 g CSFTE were obtained, respectively.

### 2.3. Inhibitory Assays of C. speciosa Fruits Extracts on XOD Activity In Vitro

The XOD activity was quantified spectrophotometrically by monitoring the production of UA from xanthine, following a previously established protocol [24]. Mixture solutions were prepared in a buffer solution containing phosphate (0.01 M) at pH 7.5 and a constant concentration of XOD (0.01 units/mL; 1 unit converts 1.0 μmol xanthine to UA per minute at 25 °C and pH 7.5.), and varying amounts of *C. speciosa* fruits extracts were exposed to incubation at a temperature of 25 degrees Celsius for a period lasting 15 min. The reaction was started by introducing substrate xanthine (9 mM) into the solution mixture. Following a 15 min incubation period, the reaction was halted by incorporating 50 μL of hydrochloric acid with a concentration of 1 N [25]. The mixture solutions were subjected to optical density (OD) measurements at 290 nm. The positive control, AP, was utilized for comparative analysis. The inhibitory potential of XOD was assessed using the following equation:

$$\mathrm{Inhibition}\;\mathrm{ratio}\;\left(\%\right)=\left(1-\frac{S-S_{0}}{B-B_{0}}\right)\times 100\%$$

The experiments conducted on the sample with and without XOD are denoted as S and S_0_, respectively. The enzyme activity without the sample is represented by B, while B_0_ refers to the control of B in the absence of both the sample and XOD.

### 2.4. Animals in Experimental Design

#### 2.4.1. Animal Ethics and Preparation

The male Sprague-Dawley rats, weighing between 180 and 220 g, were obtained from the Laboratory Animal Center of Anhui Medical University. They were given a one-week acclimation period in controlled laboratory conditions, where they had unlimited access to food and water as well as controlled temperature (25 ± 1 °C) and humidity (60 ± 10%), prior to the commencement of the experiment. The animal experimental procedures underwent evaluation and authorization from the Institutional Animal Experimentation Committee of Anhui Medical University (LLSC20232241).

#### 2.4.2. Acute Oral Toxicity Assessment

The evaluation of toxicity was carried out following the protocols established by the OECD (Organization for Economic Co-operation and Development). A total of ten rats were assigned randomly to two groups, specifically referred to as group A and B. Group A was administered a solitary oral dosage of CSFTE at a concentration of 2000 mg per kilogram of body weight, while group B was administered an elevated dosage of CSFTE, administered orally as a single dose at 5000 mg/kg body weight due to the low toxicity or non-toxicity of *C. speciosa* fruits, which can be used both as medicine and food. The rats were subjected to a 3 h fasting period prior to the administration of botanical extracts, in accordance with the methodology developed by Aliyu and colleagues [26]. Afterwards, the animals were provided with unlimited availability to regular rat chow and purified water until the completion of the experiment.

After the administration of CSFTE, the rats underwent intensive monitoring for the first 4 h, followed by daily observations over a period of 2 weeks. The parameters under observation include behavioral cues such as restlessness, lack of energy, and discomfort, respiratory patterns, ocular manifestations, cutaneous conditions, gastrointestinal disturbances, mortality rates, and any other indications of toxicity. The body weight of each individual animal was assessed using digital weighing scales on a daily basis for a duration of two weeks.

#### 2.4.3. Anti-Hyperuricemia Activity of CSFTE in the Rat Model

Preliminarily, rats were randomized into 6 groups (n = 6): normal control (NC), hyperuricemic model control (MC), AP (10 mg/kg), and CSFTE groups with 250, 500, and 1000 mg/kg, respectively. We employed AP as the positive control in our study [27]. The conventional method was employed to induce hyperuricemia in rats, which is widely acknowledged as the established animal model for conducting research on hyperuricemia [6,28]. Briefly, rats were orally administered with 100 mg/kg of HX (a precursor of uric acid) and intraperitoneally treated with 300 mg/kg of PO (an inhibitor of uricase) at 1 h prior to the administration of CSFTE. AP was utilized as specimens with known positive outcomes. The NC group of rats were orally and intraperitoneally administered with equivalent volumes of a 0.5% sodium carboxymethyl cellulose solution, mirroring the administration protocol used for the MC group. Each group of rats received daily treatment for a duration of two weeks. Intragastric administration was used for the AP control group receiving a dosage of 10 mg/kg. In the case of CSFTE groups, oral administration was employed using three different dosages (250, 500, and 1000 mg/kg) determined through prior experimentation. The rats in the NC and MC groups received an oral administration of a physiological saline solution (0.9%) at an equal dosage.

After a period of fasting, all of the animals were administered anesthesia one hour after receiving drug supplementation on the 14th day. The abdominal aorta was used to obtain blood samples, which were subsequently centrifuged at a temperature of 4 °C for 10 min with a rotational speed of 3000 revolutions per minute (rpm). The resulting serum was separated and stored in separate portions at −80 °C for future biochemical analyses. Subsequent to the blood collection process, the kidneys were promptly extracted and rinsed. Relevant kidney samples were separated into two sections to conduct histopathological assessment and Western blot analysis, correspondingly.

### 2.5. Biochemical Assay

The levels of serum UA, CRE, and BUN were determined by following the manufacturer’s instructions provided with the assay kits. In brief, the serum sample was mixed with separate detection solutions containing UA, CRE, and BUN reagents, followed by incubation at a specific temperature for a specified duration. Subsequently, the concentrations of UA, CRE, and BUN were measured using a microplate reader programmed with specific wavelengths.

### 2.6. Western Blot

The rat kidney sample weighing approximately 50 mg was carefully weighed and subsequently rinsed with saline solution at a temperature of 4 °C. Next, the tissues underwent dissection and were subsequently homogenized in a solution of RIPA buffer containing PMSF and phosphatase inhibitors. The mixture obtained was subsequently centrifuged at a speed of 12,000 revolutions per minute for a period of 10 min while maintaining a temperature of 4 °C. The liquid portion was gathered for subsequent analysis using Western blotting. A total of 6 protein samples (equivalent to 6 μL) were subjected to separation using a 10% SDS-PAGE system. Subsequently, the separated proteins were transferred onto a PVDF membrane obtained from Millipore in Burlington, MA, USA. In order to minimize non-specific binding, a solution consisting of TBST (Tris-buffered saline with 0.1% Tween-20) and 5% skimmed milk was utilized to block the membranes. Following this, the specific primary antibodies were diluted in TBST and incubated overnight with the following membranes: URAT1 (800:1), GLUT9 (10,000:1), OAT1 (2500:1), OAT3 (1000:1), and GAPDH (5000:1). Afterward, the membranes were exposed to secondary HRP-labeled goat antibody against rabbit IgG or secondary HRP-labeled goat antibody against mouse IgG antibodies for an hour. Eventually, the chemiluminescence method using ECL (Glpbio, Montclair, CA, USA) was employed to acquire the images, which were subsequently quantified utilizing ImageJ V1.8.0 software (Bio-Rad, Hercules, CA, USA).

### 2.7. H&E Staining

The renal tissues of rats were preserved in a 10% formalin solution with a neutral pH and subsequently dehydrated through a gradual increase in ethanol concentrations. After the process of dehydration, the kidney tissues were immersed in paraffin, sliced into sections measuring 4 μm thickness, and prepared for staining using hematoxylin and eosin (H&E) [29]. Subsequently, the renal sections were examined using a light microscope at a 400× magnification.

### 2.8. Statistical Analysis

The data analysis was conducted utilizing GraphPad Prism 9.3 software. Statistical significance was assessed through a one-way ANOVA, with a threshold set at *p* < 0.05 to determine the significance level.

## 3. Results

### 3.1. Inhibitory Properties of C. speciosa Fruits Extracts on XOD Activity In Vitro

**Figure 1:**
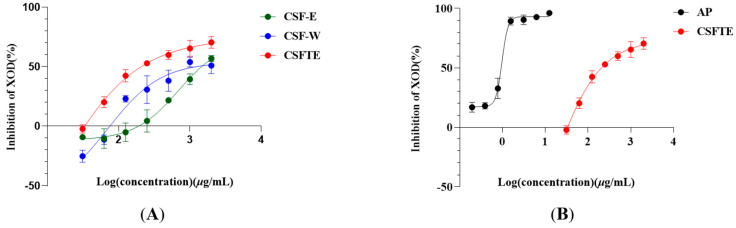
*C. speciosa* fruits extract (**A**)- and AP (**B**)-inhibited XOD in vitro. Values are expressed as means = SD.

Figure 1 shows the inhibitory effects of CSF-E, CSF-W, CSFTE, and AP on the activity of XOD. The activity of XOD was dose-dependently inhibited by four inhibitors. The CSF exhibited IC50 values for the inhibition. The values of IC_50_ of CSF-E, CSF-W, CSFTE, and AP were 1483, 1085, 334.2, and 0.8832 μg/mL, respectively. The findings indicate that CSFTE exhibited superior inhibitory effects on XOD in comparison to CSF-E and CSF-W (Figure 1A) Therefore, in vivo animal experiments with CSFTE will be conducted. Although compared with properties of *C. speciosa* fruits extracts on XOD activity in vitro, Figure 1B demonstrates that AP exhibits a relatively higher inhibitory rate on XOD. However, AP treatment can cause a series of adverse effects, such as reactions of the skin, which are considered the primary adverse effects, hematological reactions, diarrhea, and fever [30].

### 3.2. Acute Oral Toxicity Effects of CSFTE

**Figure 2:**
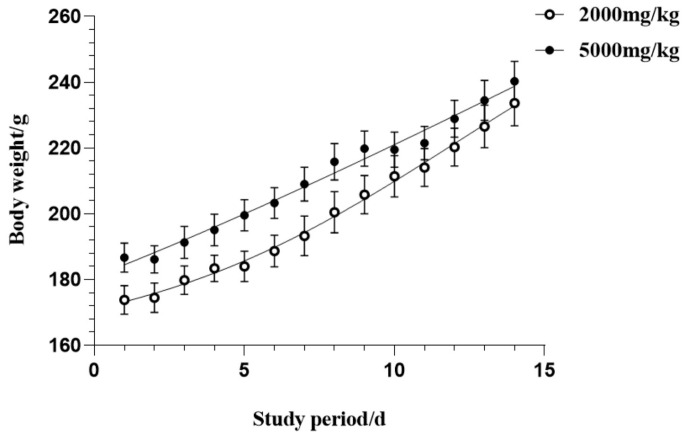
Acute oral toxicity effects of CSFTE on body weight after 14-day observation. Values are expressed as means = SEM.

In the present study, Sprague-Dawley rats of the male gender were given a solitary dosage via oral administration of CSFTE at 2000 mg/kg and 5000 mg/kg to assess potential toxicity effects. Throughout the duration of the experiment, no mortality or toxic symptoms were observed throughout the entire duration. No significant alterations in behavioral or physiological states were detected in the groups treated with the CSFET. The effect of orally administering CSFTE on the overall increase in body weight during the entire duration of the research is illustrated in Figure 2. The results demonstrate no effects of statistical significance, suggesting that CSTFE exhibits a favorable safety profile and lacks toxicity.

### 3.3. Effects of CSFTE on Biochemical Parameters

**Figure 3:**
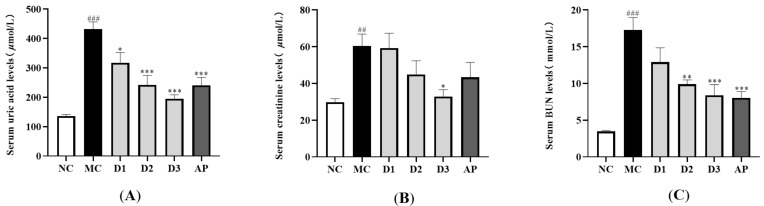
Effects of CSFTE on serum UA (**A**), CRE (**B**), and BUN (**C**) levels in the hyperuricemic rats. N = 6 per group. Values are expressed as means = SEM. NC, normal control group; MC, model control group; AP, allopurinol (10 mg/kg) group; D1, D2, and D3 mean 250, 500, and 1000 mg/kg CSFTE, respectively. ^##^ *p* < 0.01 and ^###^ *p* < 0.001 compared with the NC group; * *p* < 0.05, ** *p* < 0.01, and *** *p* < 0.001 compared with the MC group.

Figure 3A illustrates the impact of CSFTE on levels of serum UA. Following the administration of HX and PO, there was a notable rise observed in the serum UA levels within the MC group (*p* < 0.001), suggesting effective induction of hyperuricemia in rats. When compared to the MC group, CSFTE exhibited a notable influence on hyperuricemic rats. The administration of CSFTE at doses of 250, 500, and 1000 mg/kg resulted in a dose-dependent decrease in serum UA levels (*p* < 0.05, *p* < 0.001, and *p* < 0.001, respectively). These results indicate that CSFTE possesses evident anti-hyperuricemic properties.

Figure 3B shows a notable disparity observed in the serum CRE levels between the MC group and NC group (*p* < 0.01). The levels of CRE were slightly reduced in the CSFTE group (500 mg/kg) and AP group (10 mg/kg) compared to the MC group. Meanwhile the doses of 1000 mg/kg CSFTE showed significant difference (*p* < 0.05), which means less renal injury.

The presence of renal dysfunction can be indicated by the level of BUN. As depicted in Figure 3C, the NC group exhibited a lower BUN level compared to the MC group (*p* < 0.001). Significantly decreased serum BUN levels were observed in the groups administered with CSFTE at doses of 500 and 1000 mg/kg, compared to the MC group (*p* < 0.01 and *p* < 0.001, respectively). The AP group (10 mg/kg) showed a significant difference (*p* < 0.001). The dose of 250 mg/kg CSFTE showed no significant difference but with lower BUN levels than the MC group, which means less renal injury.

### 3.4. CSFTE Down-Regulated Renal Protein Levels of URAT1 and GLUT9 and Up-Regulated Renal Protein Levels of OAT1 and OAT3 in Hyperuricemic Rats

**Figure 4:**
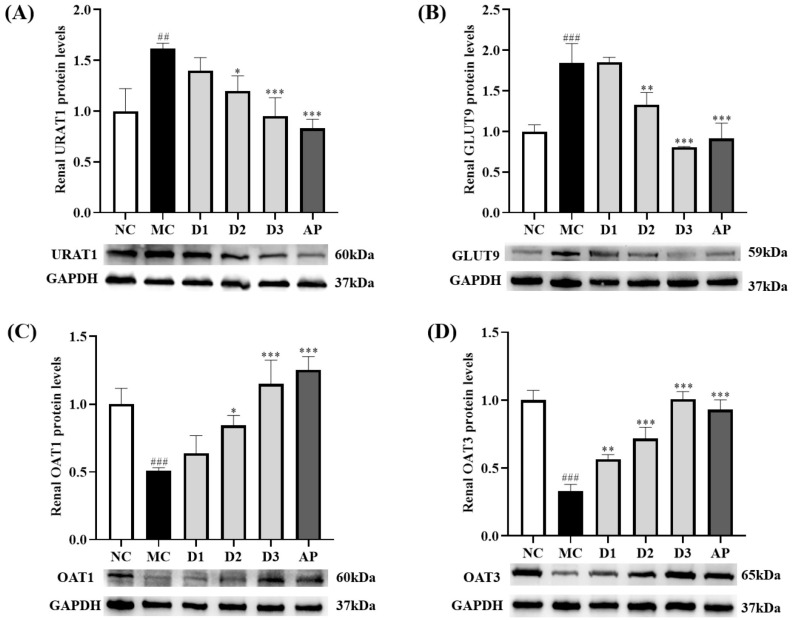
Effects of CSFTE and AP on renal URAT1 (**A**), GLUT9 (**B**), OAT1 (**C**), OAT3, and (**D**) protein levels in hyperuricemic rats. N = 6 per group. Values are expressed as means = SD. NC, normal control group; MC, model control group; AP, allopurinol (10 mg/kg) group; D1, D2, and D3 mean 250, 500, and 1000 mg/kg CSFTE, respectively. ^##^ *p* < 0.01 and ^###^ *p* < 0.001 compared with the NC group; * *p* < 0.05, ** *p* < 0.01, and *** *p* < 0.001 compared with the MC group.

To assess the influence of CSFTE on the protein expression levels of vital transporters associated with renal uric acid (UA) regulation, we performed Western blot analysis. (Figure 4, Figure S1). 

The effects of CSFTE on the expression of URAT1 and GLUT9 in rats are demonstrated in Figure 4A,B. After a 14-day oral administration of HX and PO, the MC group exhibited significantly increased levels of URAT1 and GLUT9 compared to the NC group (*p* < 0.01 and *p* < 0.001, respectively), indicating a potential influence on renal function. Following treatment with AP and CSFTE at low, middle, and high dosages, there was a decrease observed in URAT1 levels in rats compared to the MC groups. Significant differences were observed in AP and CSFTE at middle and high dosages (*p* < 0.001, *p* < 0.05, and *p* < 0.001, respectively), particularly in relation to the levels of GLUT9 in rats after treatment with AP and CSFTE at middle and high dosages (*p* < 0.001, *p* < 0.01, and *p* < 0.001, respectively) compared to the MC groups, where GLUT9 levels were significantly decreased.

Figure 4C,D shows the effects of CSETF on the expression of OAT1 and OAT3 in rats. The protein levels of OAT1 and OAT3 in the MC group of rats were lower than in the NC group of rats (*p* < 0.001 and *p* < 0.001, respectively). The administration of AP and middle/high doses of CSFTE led to a notable elevation in OAT1 levels in rats (*p* < 0.001, *p* < 0.05, and *p* < 0.001, respectively) when compared to the MC group. Although there was an upward trend observed in the low-dose CSFE group compared to the MC group, no significant difference was found. In the MC group, there was a noticeable decrease in the protein level of OAT3 compared to CSFTE at all three doses (*p* < 0.01, *p* < 0.001, and *p* < 0.001, respectively).

### 3.5. Effects of CSFTE on Improving Renal Dysfunction

**Figure 5:**
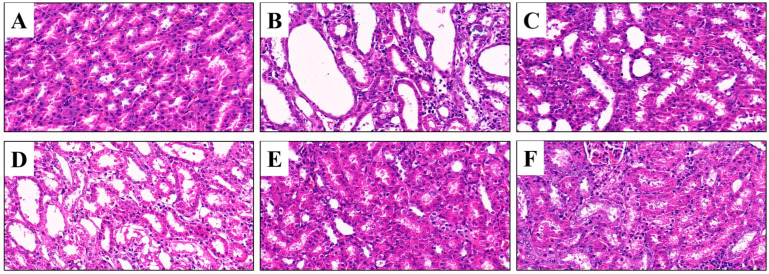
Histopathology assays of the kidney tissues (H&E, 400×). Scale bar: 20 μm. (**A**) Normal control group; (**B**) model control group; (**C**) allopurinol (10 mg/kg) group; (**D**–**F**) 250, 500, and 1000 mg/kg CSFTE, respectively.

The kidneys have a vital function in regulating UA balance, thus necessitating the imperative morphological assessment of renal tissues for comprehensive investigation of renal health in clinical trials. The rats in the NC group displayed renal tissues with unaltered morphologies (Figure 5A). The renal tissue of rats in the MC group exhibited dilation of the renal tubule lumen, inflammatory infiltration, necrosis, and exfoliation of tubular epithelial cells, indicating significant structural damage to the kidneys (Figure 5B). However, the AP group of rats did not exhibit significant improvements in renal damage (Figure 5C). In contrast, treatment with CSFTE resulted in varying degrees of attenuation of kidney damage (Figure 5D–F). Furthermore, the histopathologic sections of hyperuricemic rats showed that the administration of CSFTE at doses of 500 and 1000 mg/kg effectively protected against glomerular and kidney tubule damages.

## 4. Discussion

Clinical reports have demonstrated that HUA is the primary risk factor for gout. Furthermore, chronic nephritis, renal dysfunction, and metabolic syndromes have been found to be linked with this condition [31,32,33,34,35]. Previous studies have demonstrated that the excessive production of UA is attributed to heightened XOD activity within the organism [36]. XOD belongs to the group of enzymes known as xanthine oxidoreductases, playing a pivotal role as an essential enzyme responsible for catalyzing the oxidation process of xanthine to UA [37]. Consequently, the potential use of XOD inhibitors has been suggested as a strategy that could be employed for the purpose of preventing or treating hyperuricemia. The previous in vitro XOD inhibition experiment showed that the inhibition rate of CSFTE was better than that of CSF-E and CSF-W. Hence, we selected CSFTE for subsequent in vivo animal experiments.

AP is frequently prescribed as an XOD inhibitor for the clinical management of gout. PO is predominantly utilized for the development of an animal model of HUA through uricase inhibition [38]. The conversion of HX to xanthine and subsequent catalysis by XOD leads to the formation of UA. UA levels can be directly increased by excessive intake of HX supplements. To create a more consistent and resilient model for HUA, various studies have utilized the concurrent administration of two or three prototype medications [28,39]. Therefore, in our study, we employed PO and HX as treatment modalities. Remarkably elevated levels of UA were observed following 14 days of co-administration with HX and PO, indicating the successful establishment of the HUA model.

In this research, we have successfully illustrated that the administration of CSFTE resulted in varying degrees of efficacy in reducing serum UA levels in hyperuricemic rats. Notably, CSFTE exhibited significant hypouricemic effects and its effectiveness was clearly dependent on the dosage administered. It is worth mentioning that the levels of BUN and serum CRE serve as crucial indicators for evaluating renal function [38]. Renal impairment may be associated with increased serum levels of BUN and CRE, suggesting a decline in the elimination of urea and creatinine [40]. Compared to the MC group, treatment with CSFTE at three different doses (250, 500, and 1000 mg/kg) resulted in a reduction in serum BUN and CRE levels. This suggests that CSFTE has the potential to reverse kidney damage caused by HUA in rats. Consequently, these findings provide further evidence supporting the link between HUA and impaired renal function induced by PO with HX, while highlighting the significant role played by CSFTE in improving renal damage.

The elimination of UA is accomplished through the intricate interplay between renal reabsorption and secretion mechanisms, which involve a multitude of transporters inherent to the kidney. For example, URAT1 and GLUT9 mediate UA reabsorption by transporting UA from the lumen of the kidney to the blood [41,42]. UA is secreted from the blood to epithelial cells by OAT1 and OAT3 [42,43]. The findings of the current investigation indicate that administration of CSFTE at moderate and high doses (500 and 1000 mg/kg) results in significant reduction in renal protein levels of URAT1 and GLUT9, while simultaneously leading to a substantial increase in the levels of renal proteins OAT1 and OAT3.

In addition, renal histopathological sections showed varying degrees of attenuation in kidney damage with the administration of CSFTE. However, AP did not demonstrate significant improvements in renal damage.

It has been approximated that adverse reactions occur in around 2% of patients who undergo AP treatment [44]. But CSFTE, a naturally occurring substance, exhibits potential applications in both the medical and food industries. In contrast to AP, it is characterized by its safety profile with minimal side effects or toxicity. It has been observed that varying doses of CSFTE have the potential to lower levels of serum UA. It is possible to speculate that the decline may be linked to the metabolic processes of the human body. Additional investigation is necessary to evaluate the manifestation of pertinent urate transporter proteins, which may present as potential therapeutic targets for CSFTE in the prevention and management of HUA. In addition, the constituents of CSFTE, responsible for the reduction in UA levels, necessitate further investigation and identification.

## 5. Conclusions

**Figure 6:**
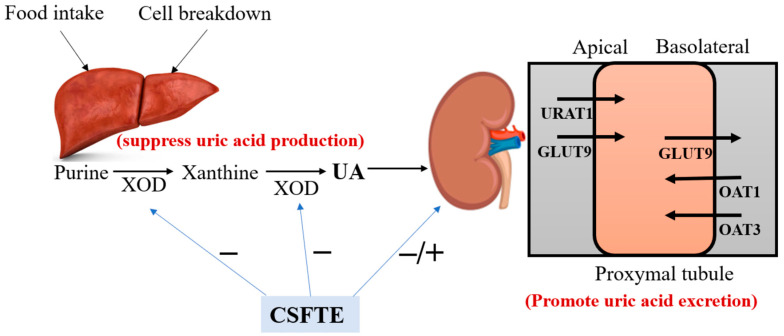
The schematic diagram of the UA pathway under the influence of CSFTE.

In summary, the hypouricemic effect of CSFTE could be explained mainly by two aspects (Figure 6). Firstly, the hypouricemic actions were achieved by CSFTE through its ability to inhibit XOD. Secondly, the rats with hyperuricemia exhibited a decrease in serum UA levels following the administration of CSFTE. CSFTE was found to regulate the expression of renal URAT1 and GLUT9 proteins, resulting in elevated levels of renal OAT1 and OAT3 protein expression. This contributed to the promotion of UA excretion by the kidneys, thereby improving renal function. Additionally, CSFTE exhibited protective effects against inflammatory cell infiltration and tubular damage observed in the histopathological sections of hyperuricemic rat kidneys. These results offer substantiation for the potential use of CSFTE as a health product or medication in the prevention and treatment of hyperuricemia-associated ailments. Additionally, they elucidate the anti-hyperuricemic and nephroprotective effects of CSFTE for managing hyperuricemia, along with its plausible mechanism.

## Data Availability

Data presented in this study are available on request from the corresponding author. Data are only available on request due to ethical restrictions.

## Supplementary Materials

The following supporting information can be downloaded at: https://www.mdpi.com/article/10.3390/metabo14020117/s1, Figure S1: The full original images of Western blots used in this study.
