# Supplementary material for: Anti-Hyperuricemic Effects of Extracts from Chaenomeles speciosa (Sweet) Nakai Fruits on Hyperuricemic Rats
Source: Metabolites. 2024 Feb 10;14(2):117. doi: 10.3390/metabo14020117 (PMC10890149; doi:10.3390/metabo14020117)

Anti-hyperuricemic effects of extracts from *Chaenomeles speciosa* (Sweet) Nakai fruits in hyperuricemic rats

Ruoling Xu, Peng Deng, Yiren Ma, Kui Li, Fucai Ren * and Ning Li *

Anhui Key Laboratory of Bioactivity of Natural Products, School of Pharmacy, Anhui Medical University, He-fei 230032, China

All the original images of Western blots were provided here, including four proteins, namely URAT1, GLUT9, OAT1, OAT3 respectively. Each protein of Western blots was repeated three times. The six lanes in all figures were represented from left to right, normal control group, model control group, 250 mg/kg CSFTE , 500 mg/kg CSFTE, 1000 mg/kg CSFTE and allopurinol (10 mg/kg) group respectively.

1. URAT1 (Representative graph)

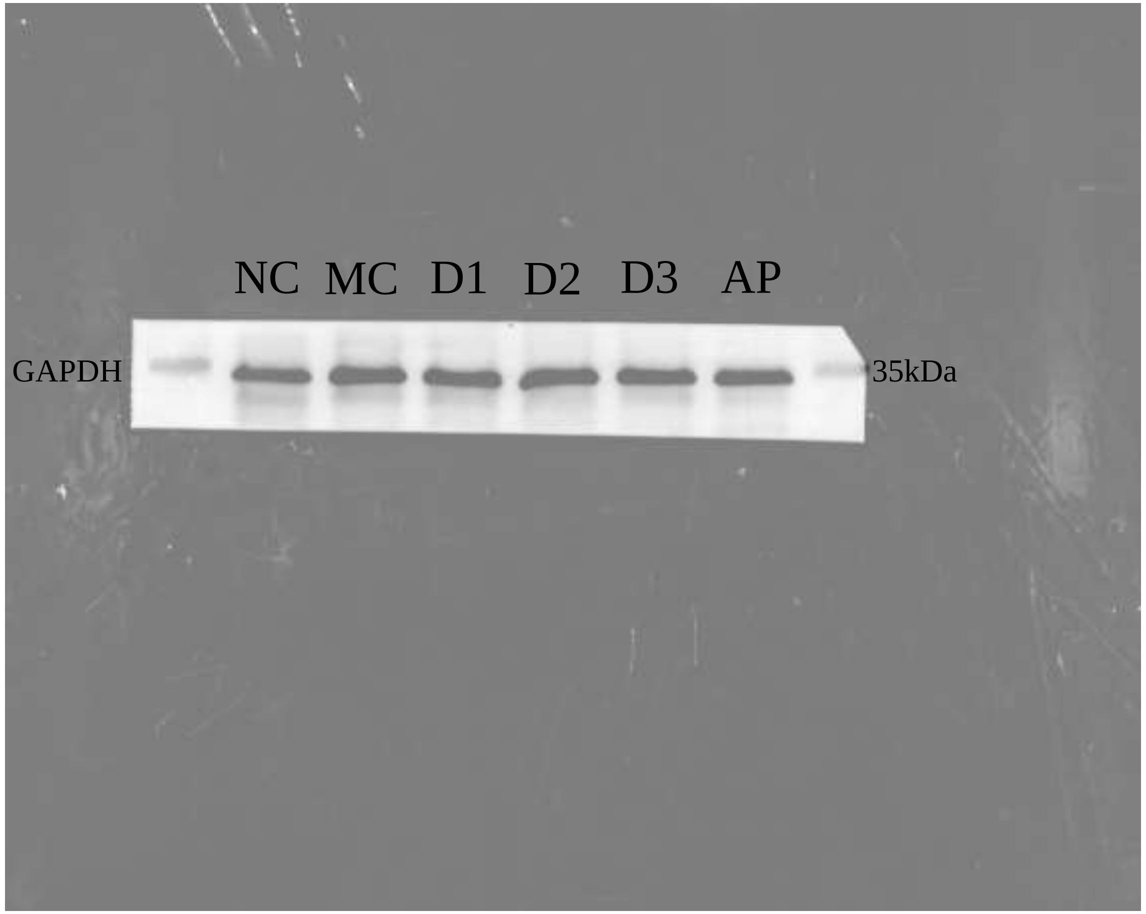

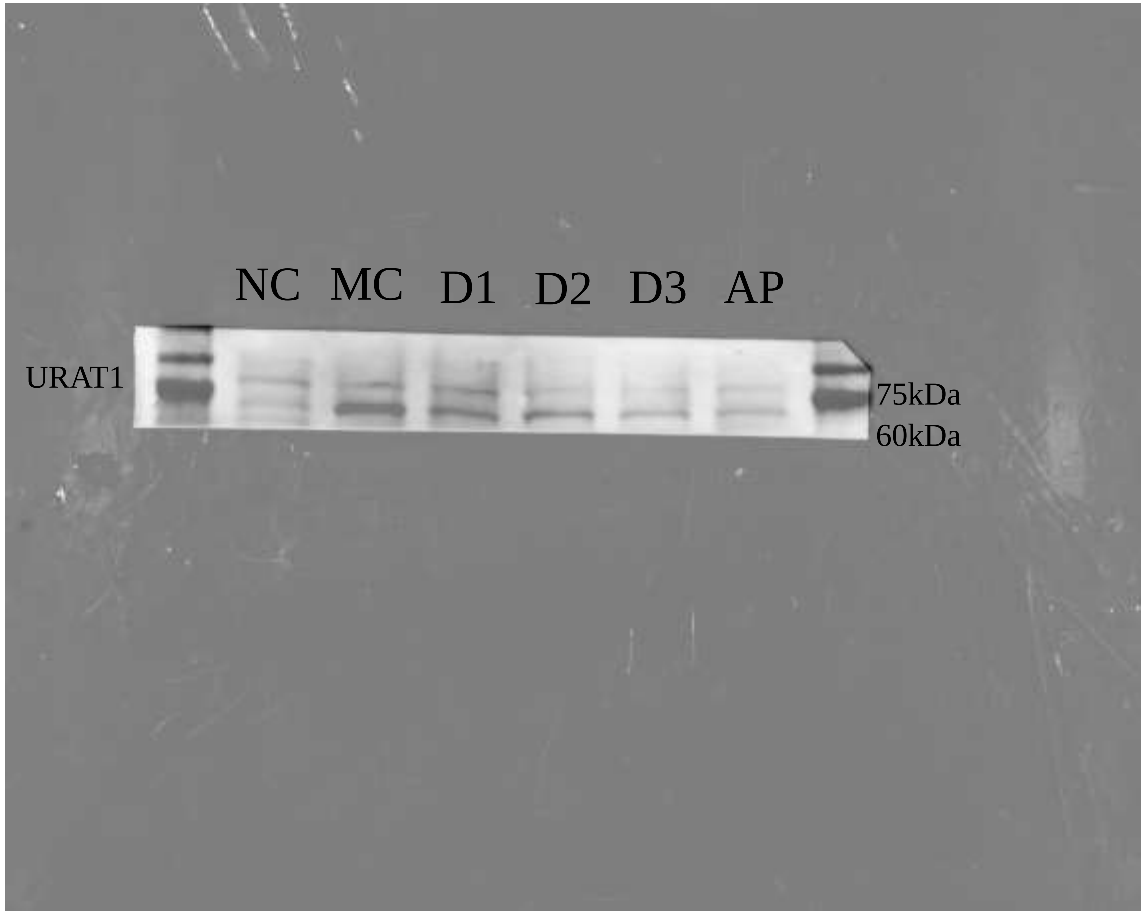

2. URAT1

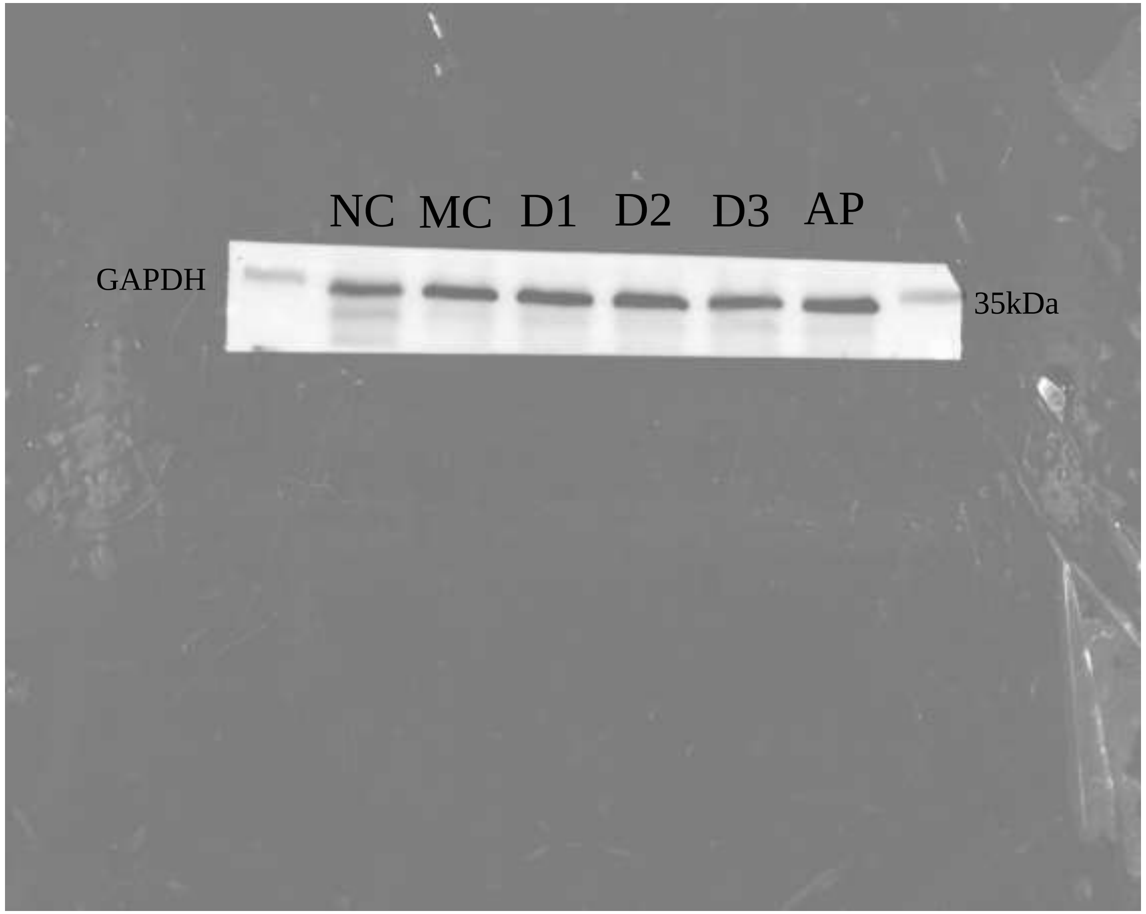

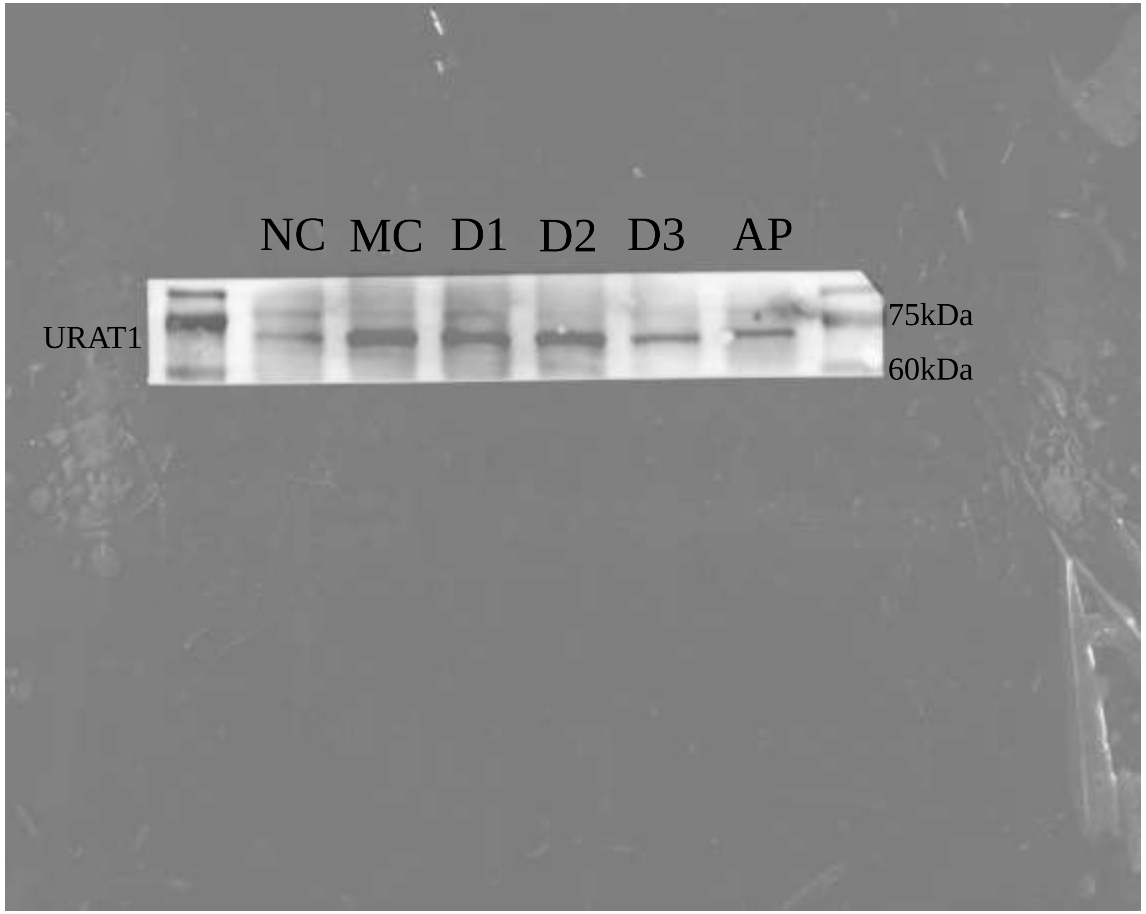

3. URAT1

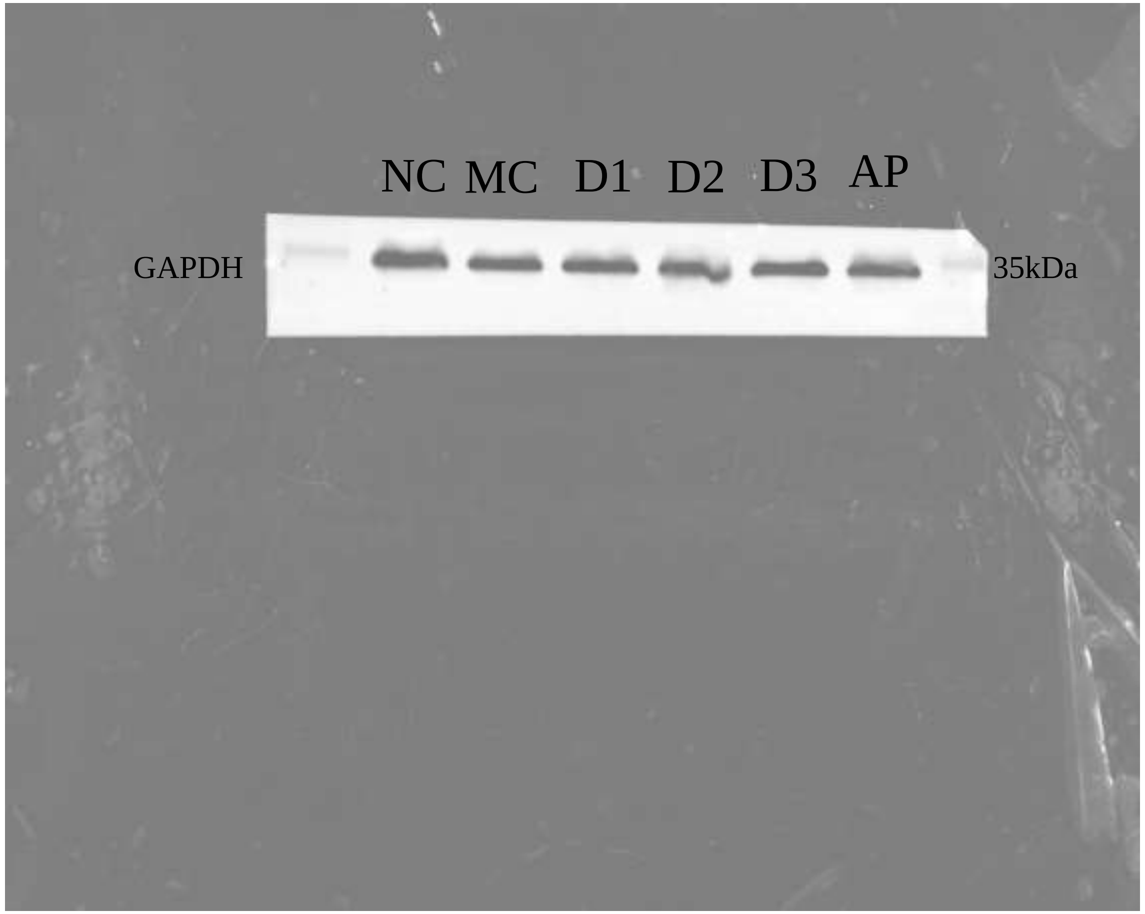

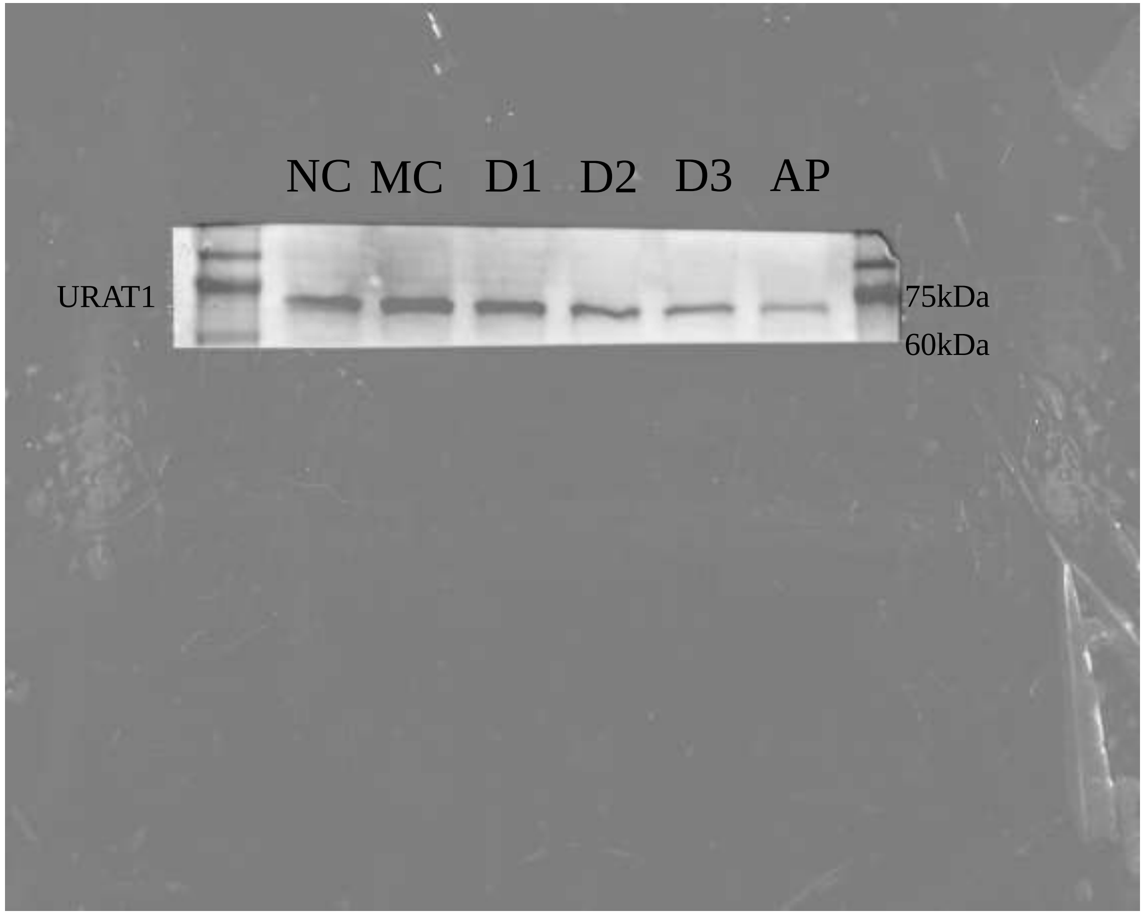

1. GLUT9 (Representative graph)

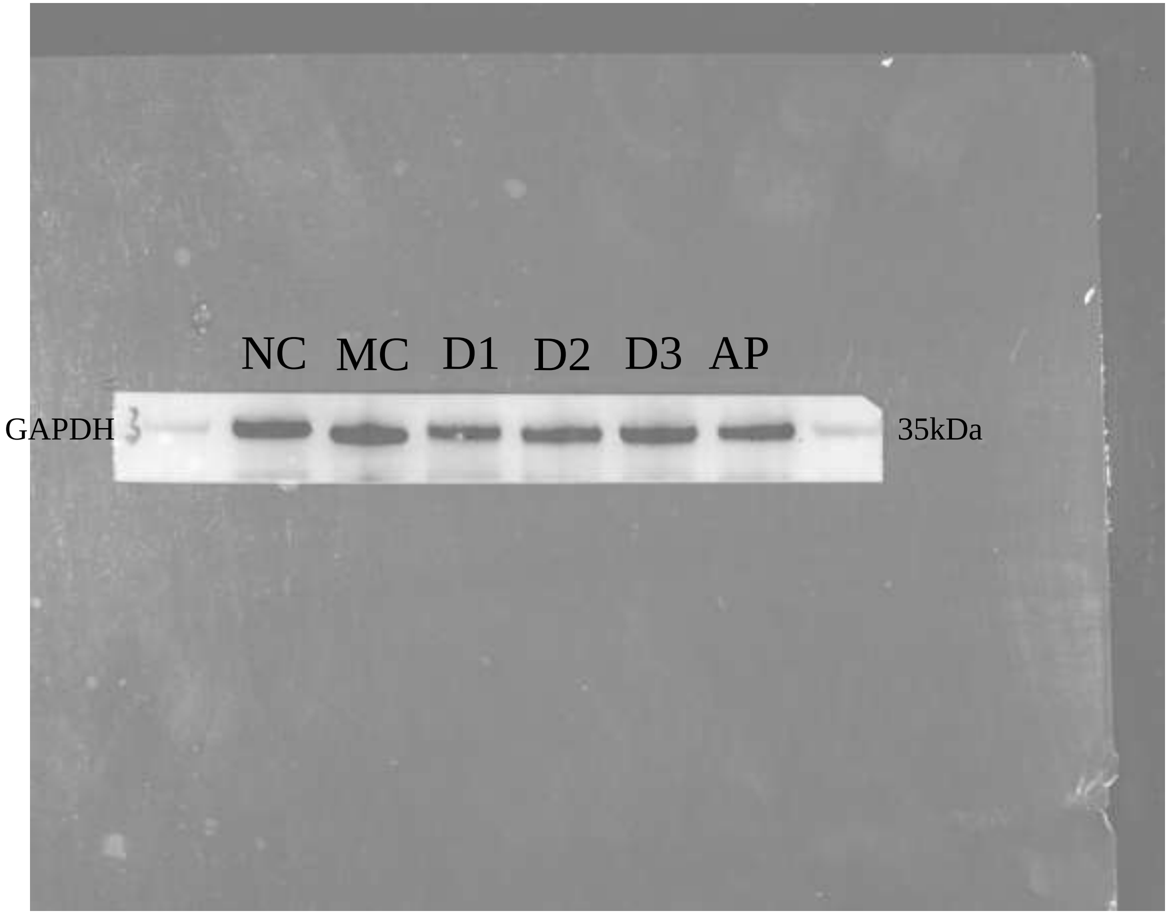

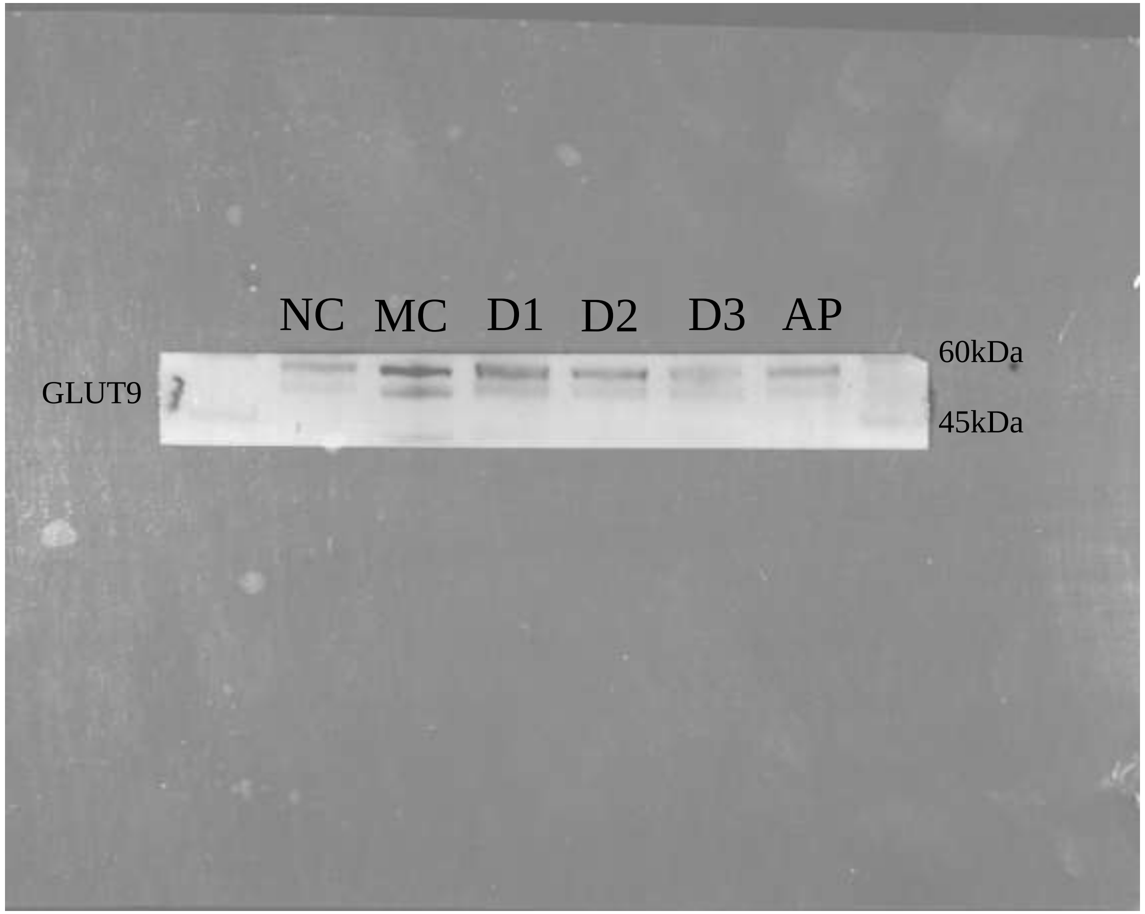

2. GLUT9

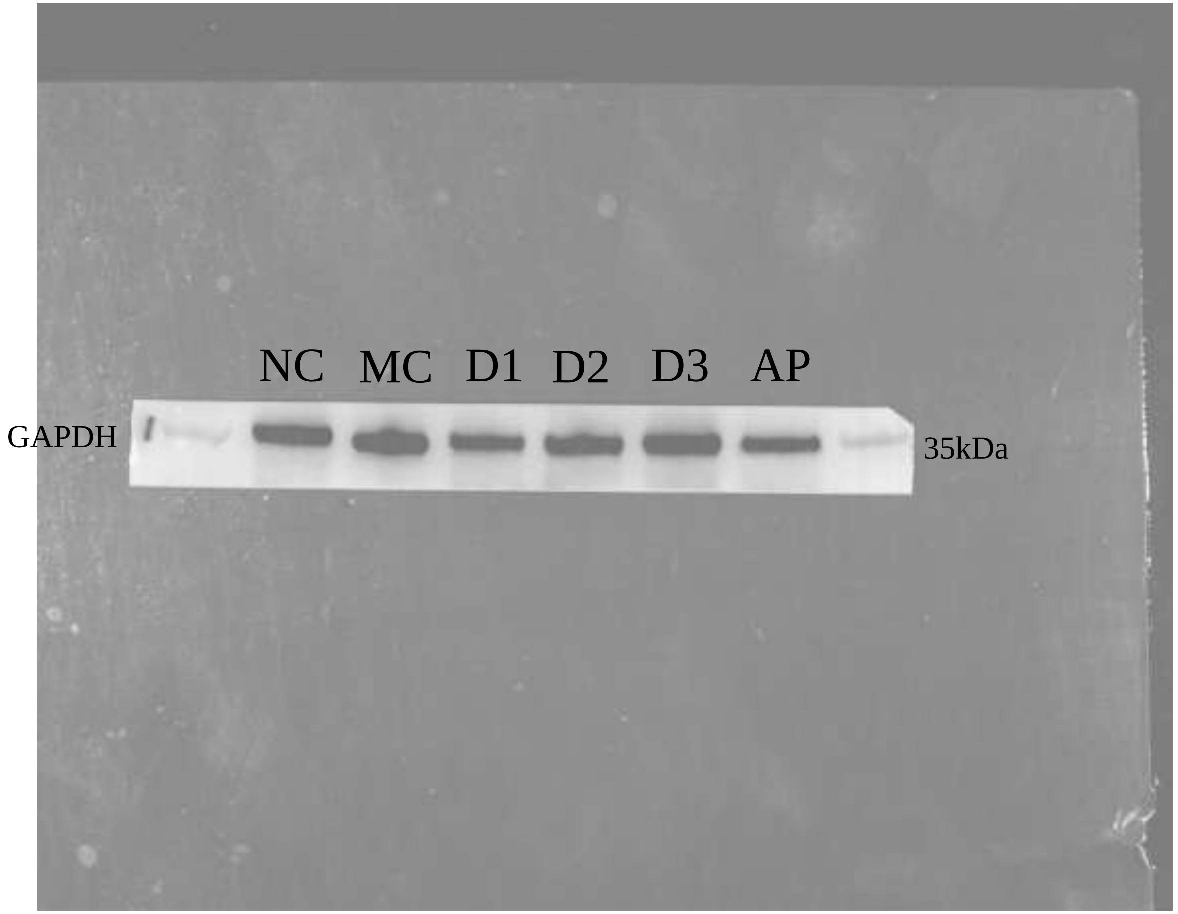

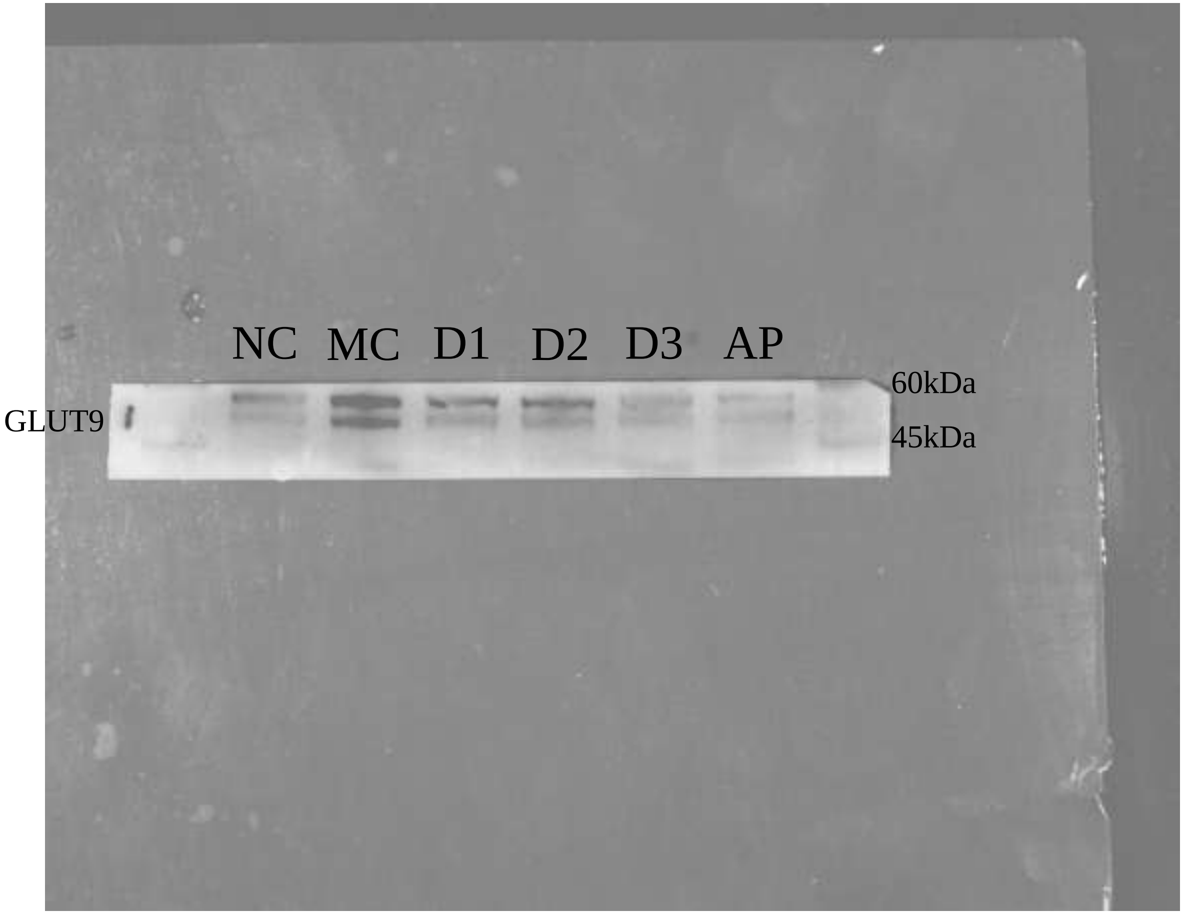

3. GLUT9

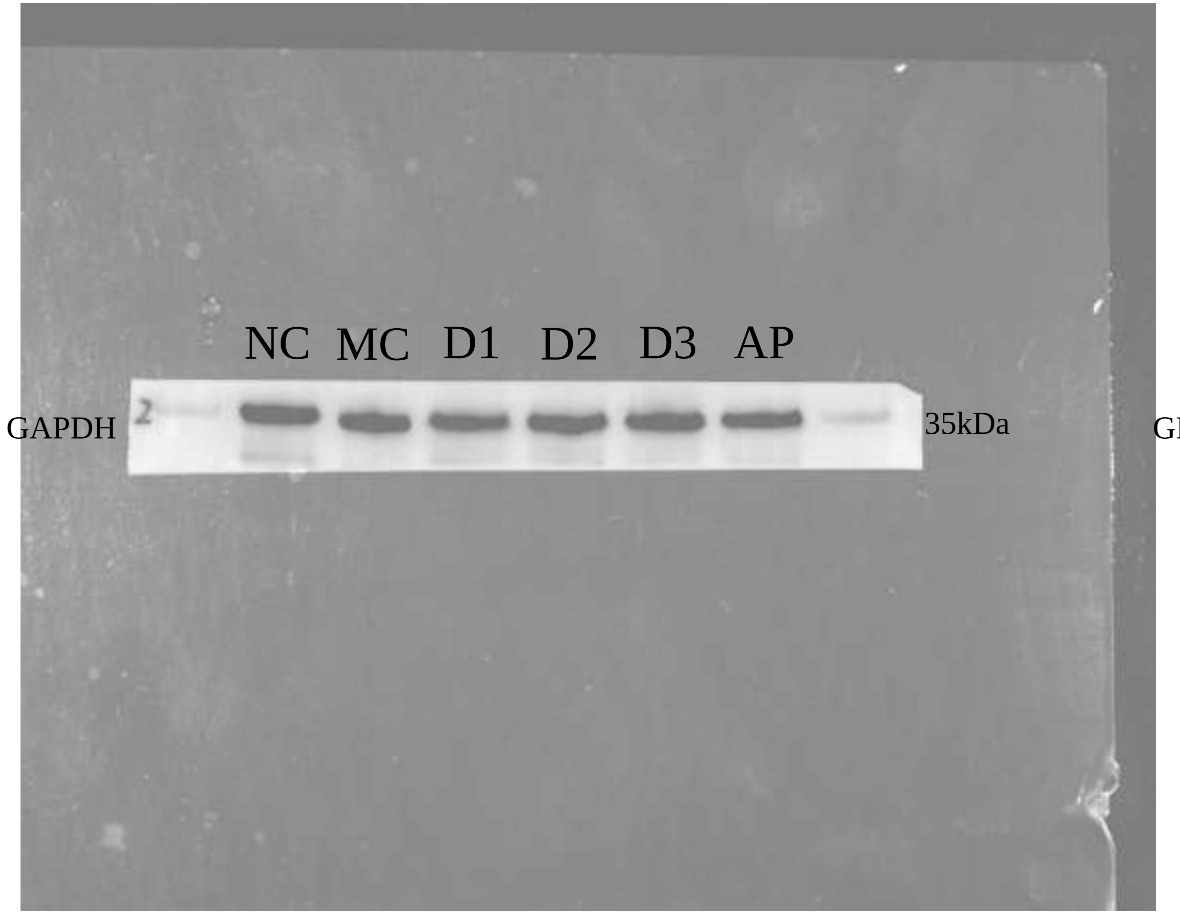

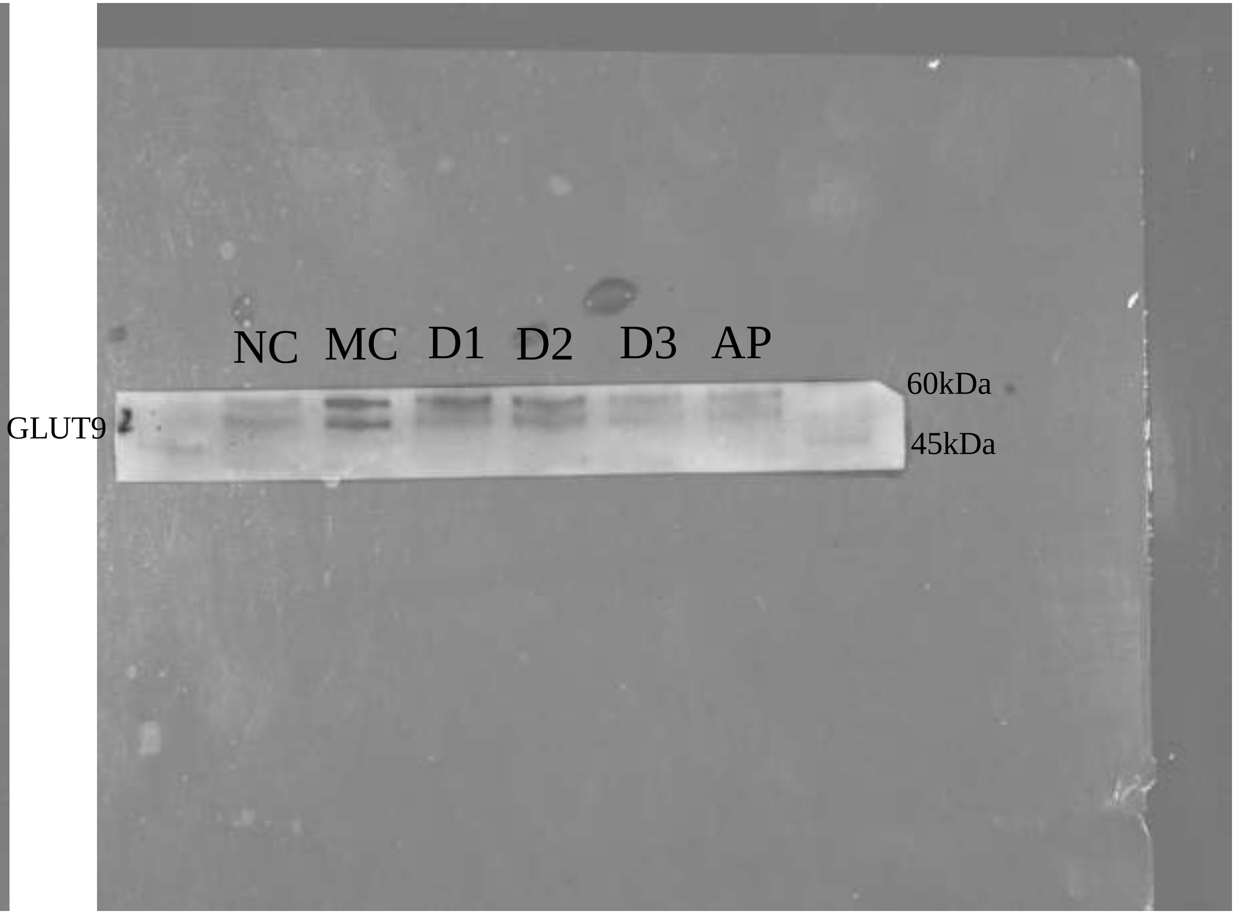

1. OAT1 (Representative graph)

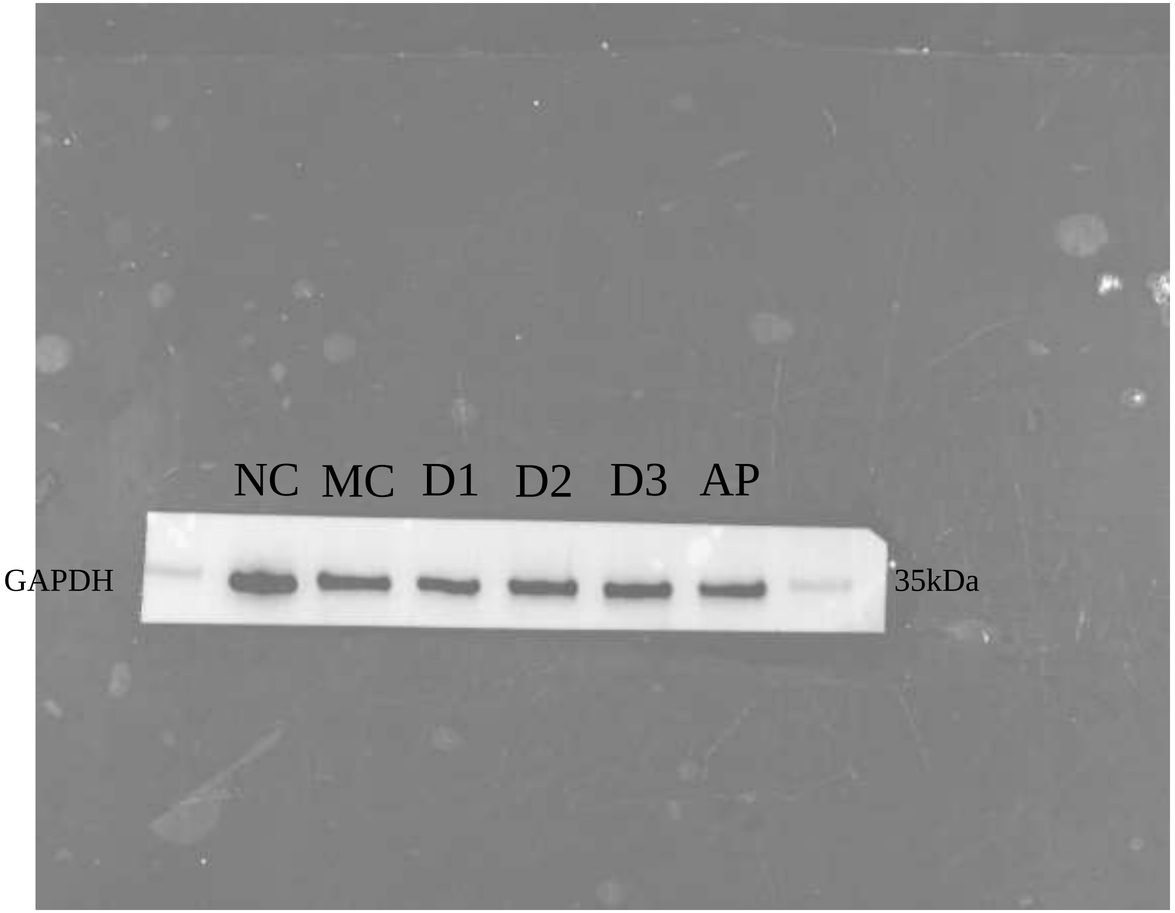

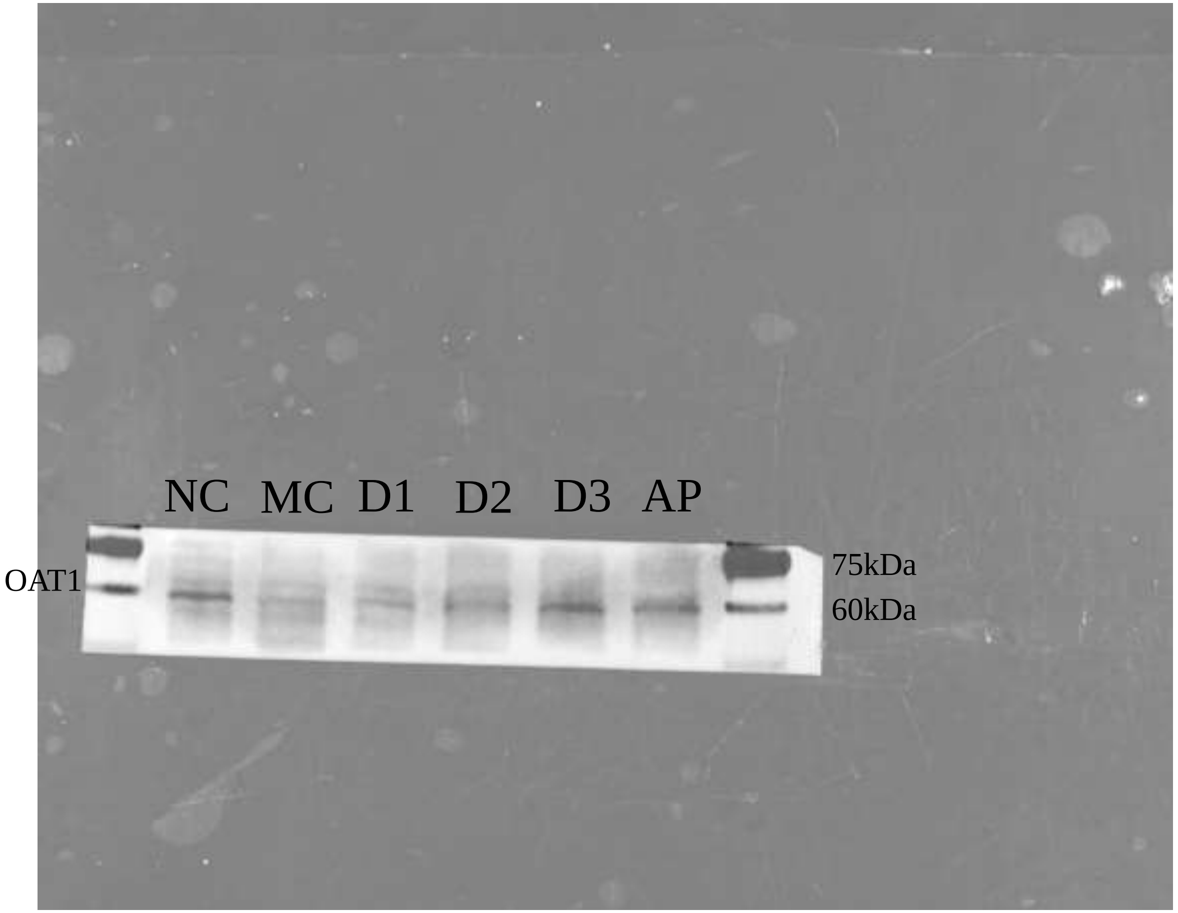

2. OAT1

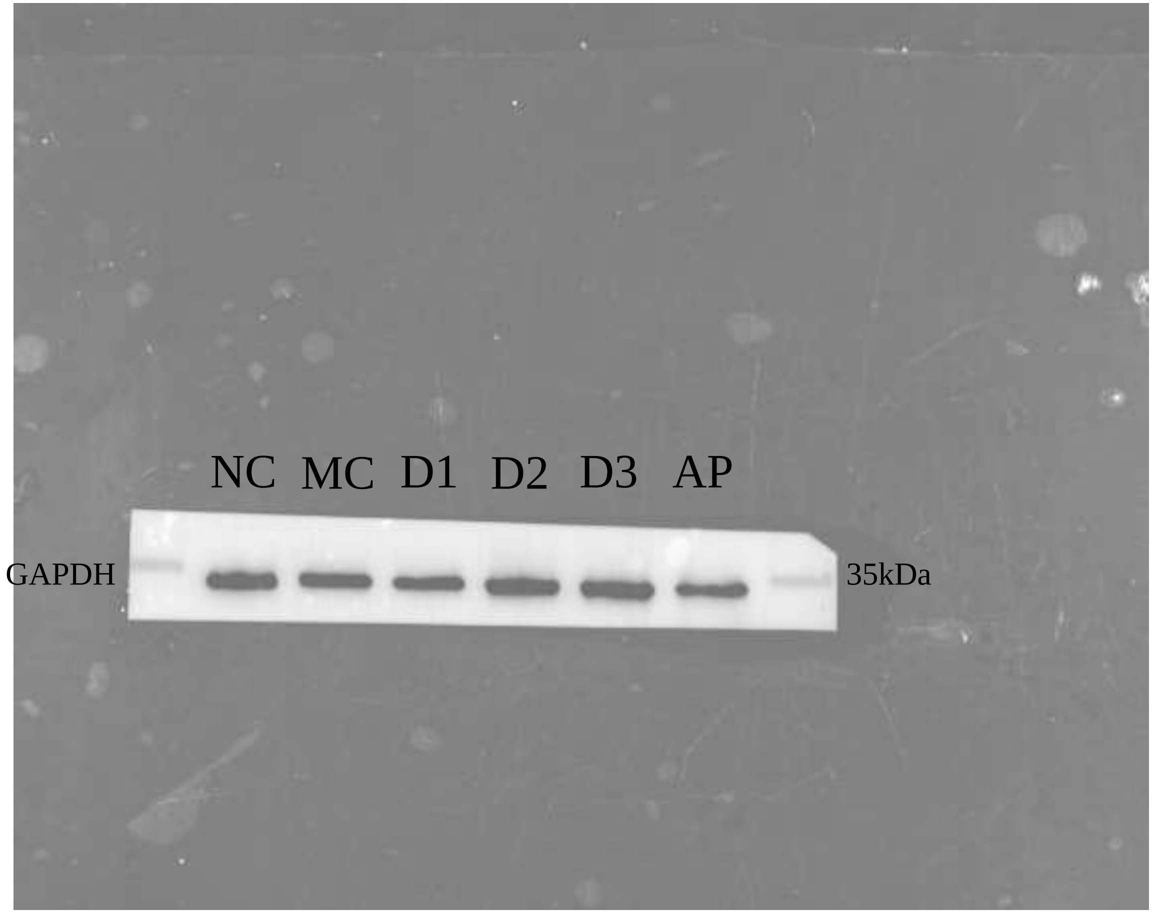

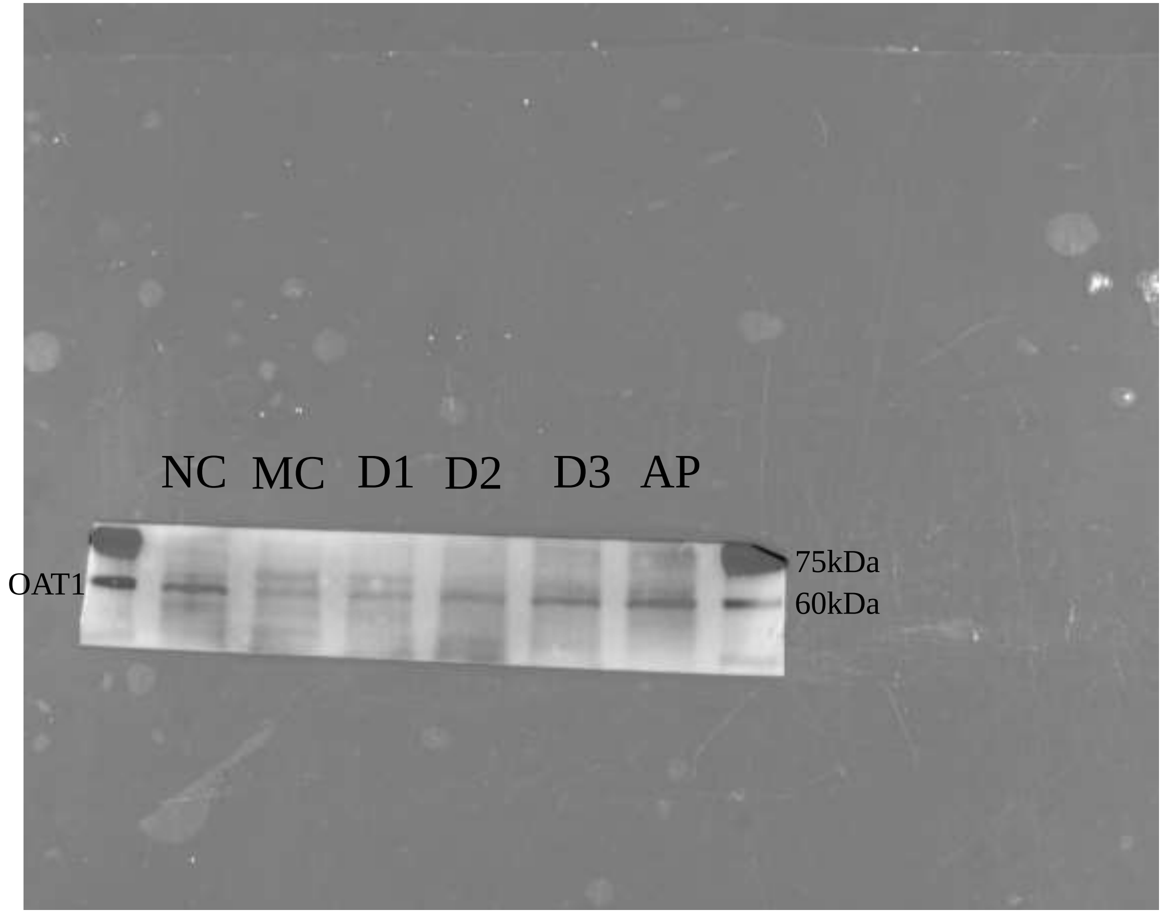

3. OAT1

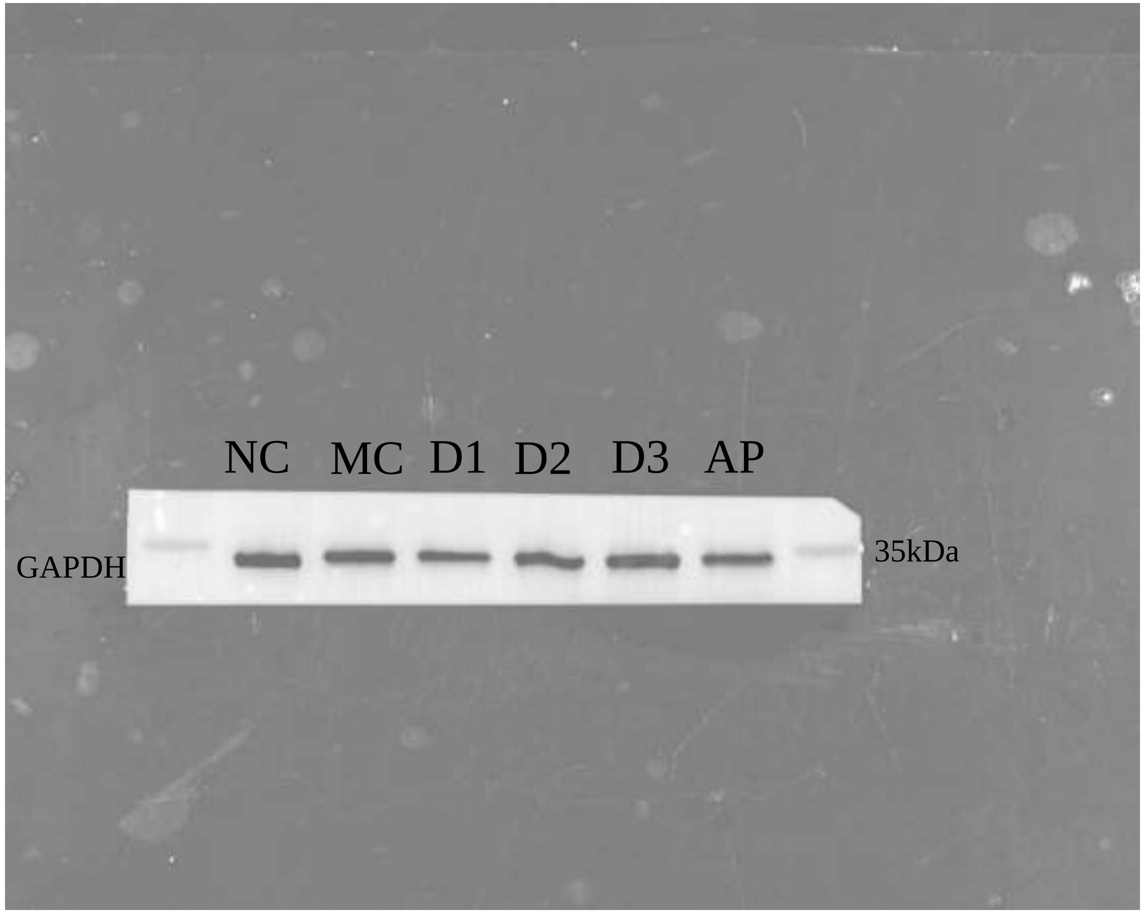

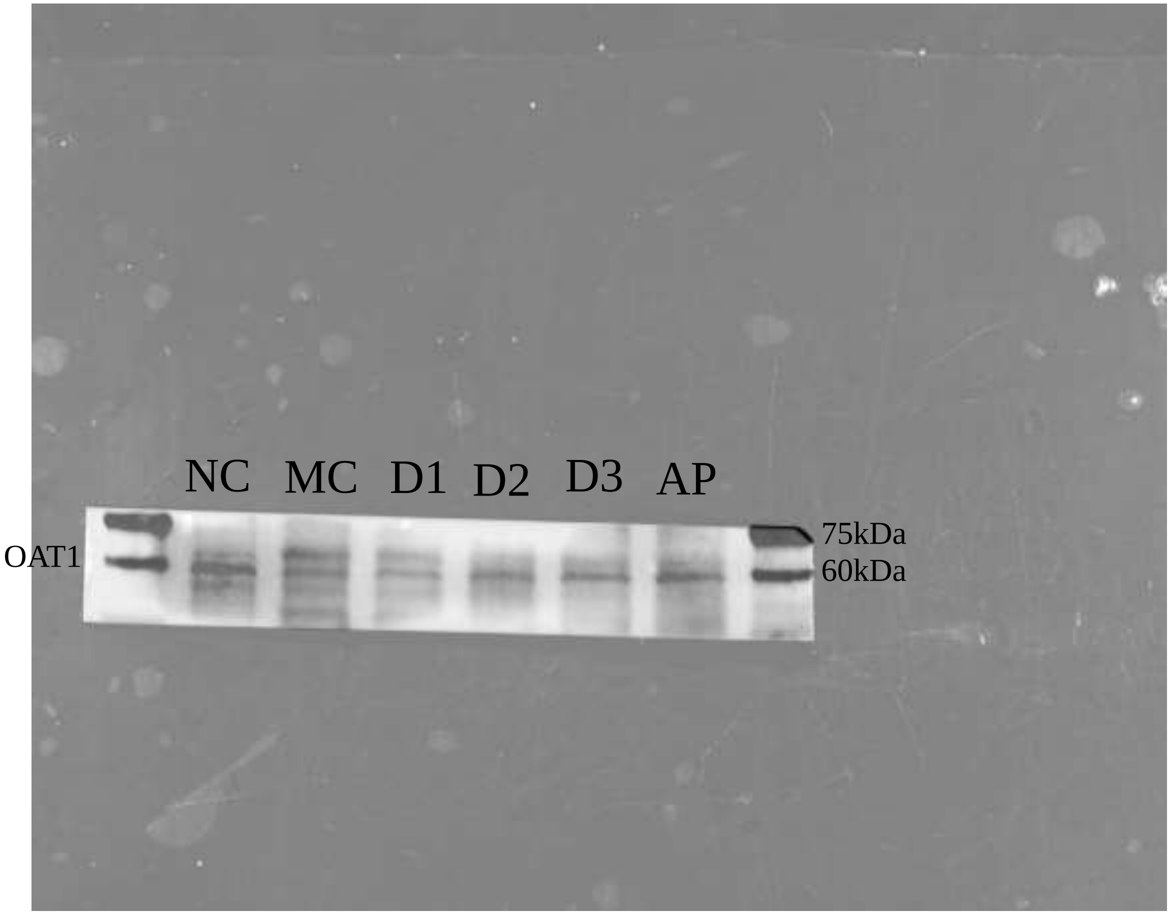

1. OAT3 (Representative graph)

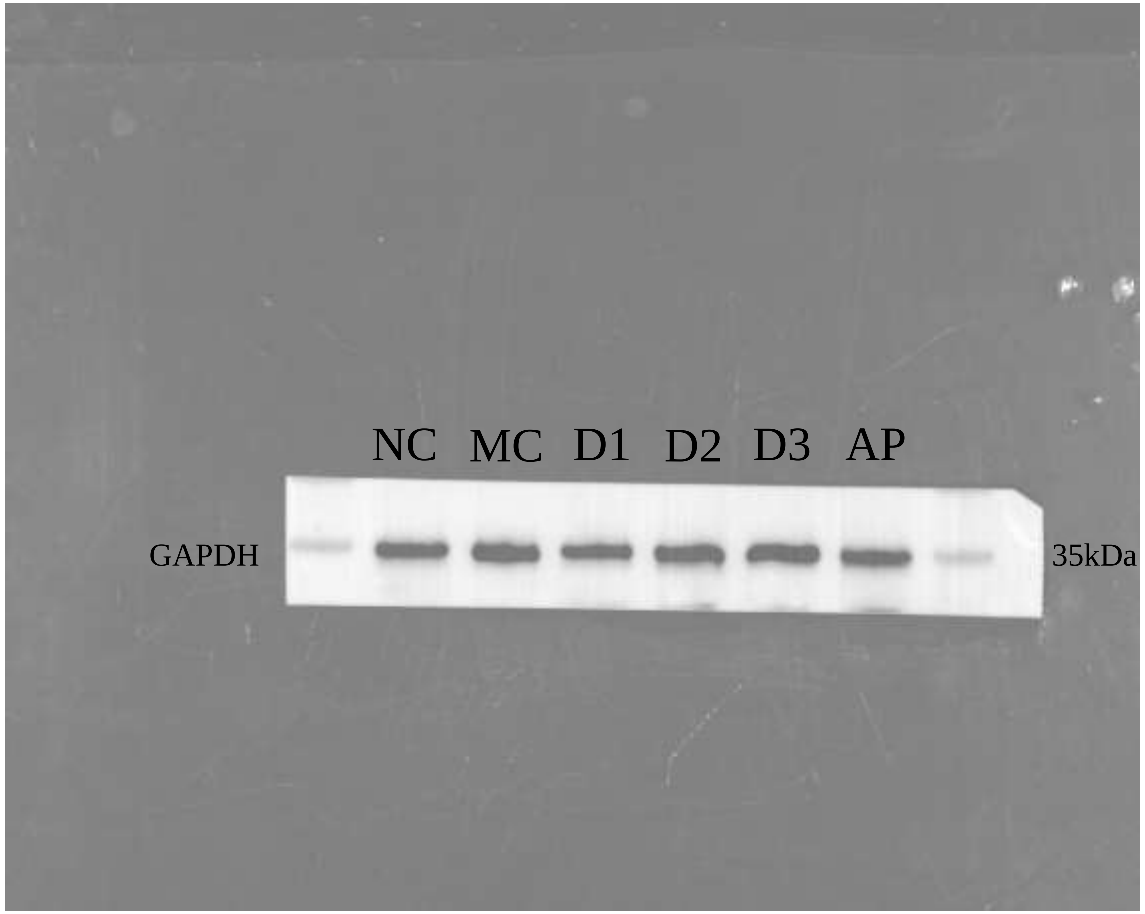

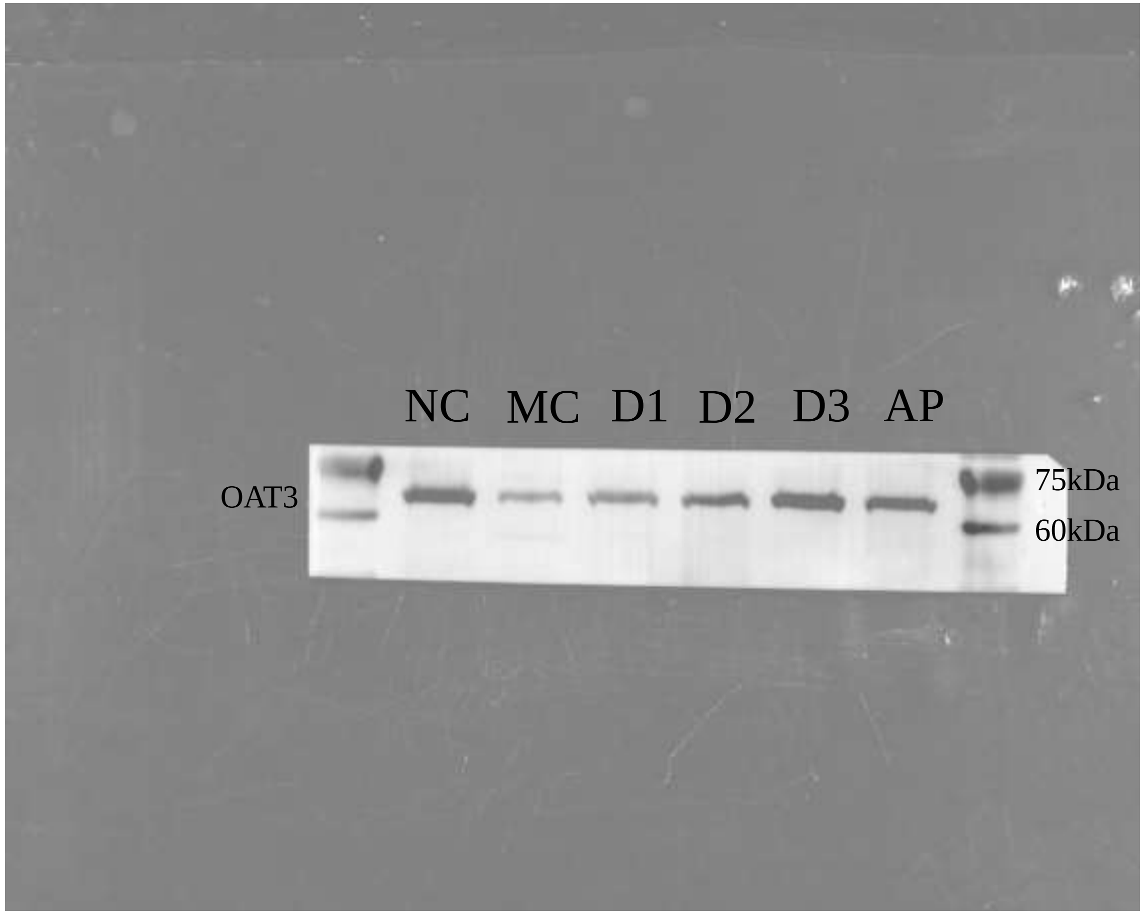

2. OAT3

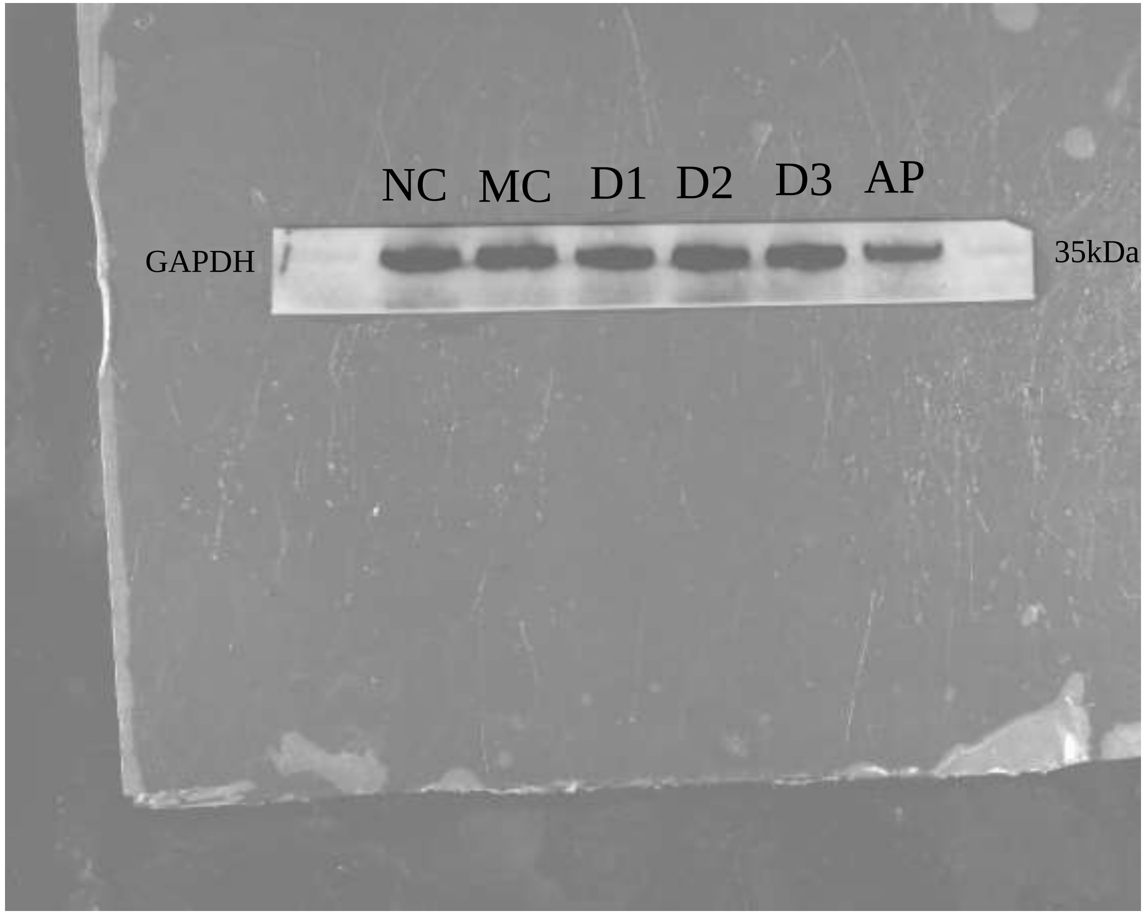

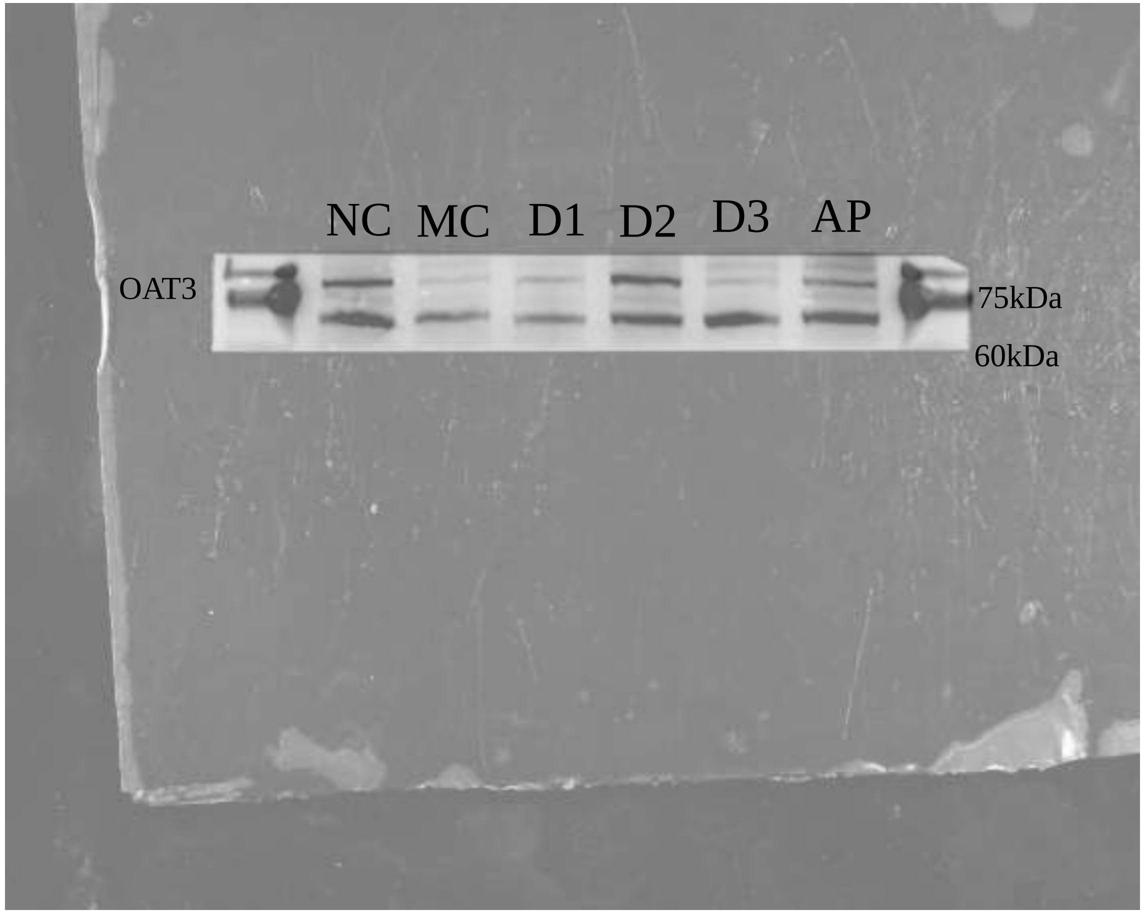

3. OAT3

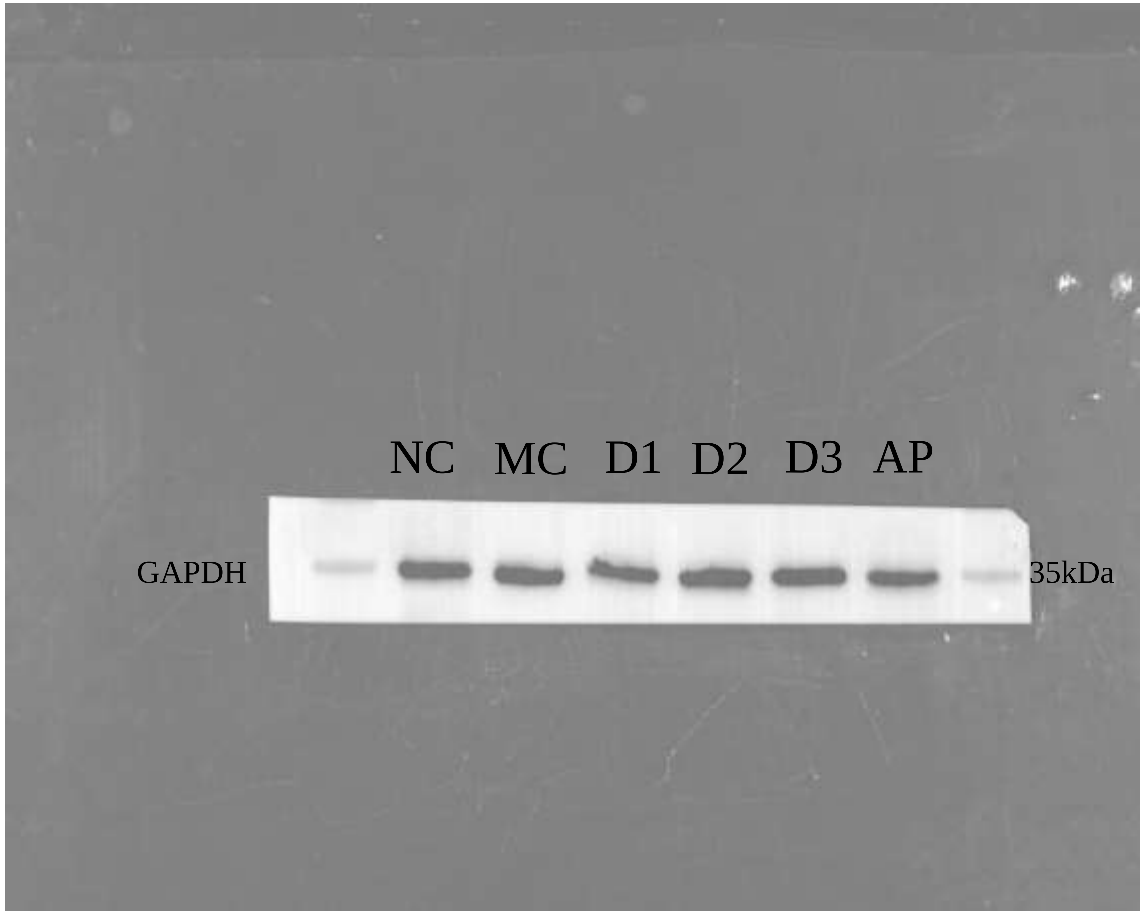

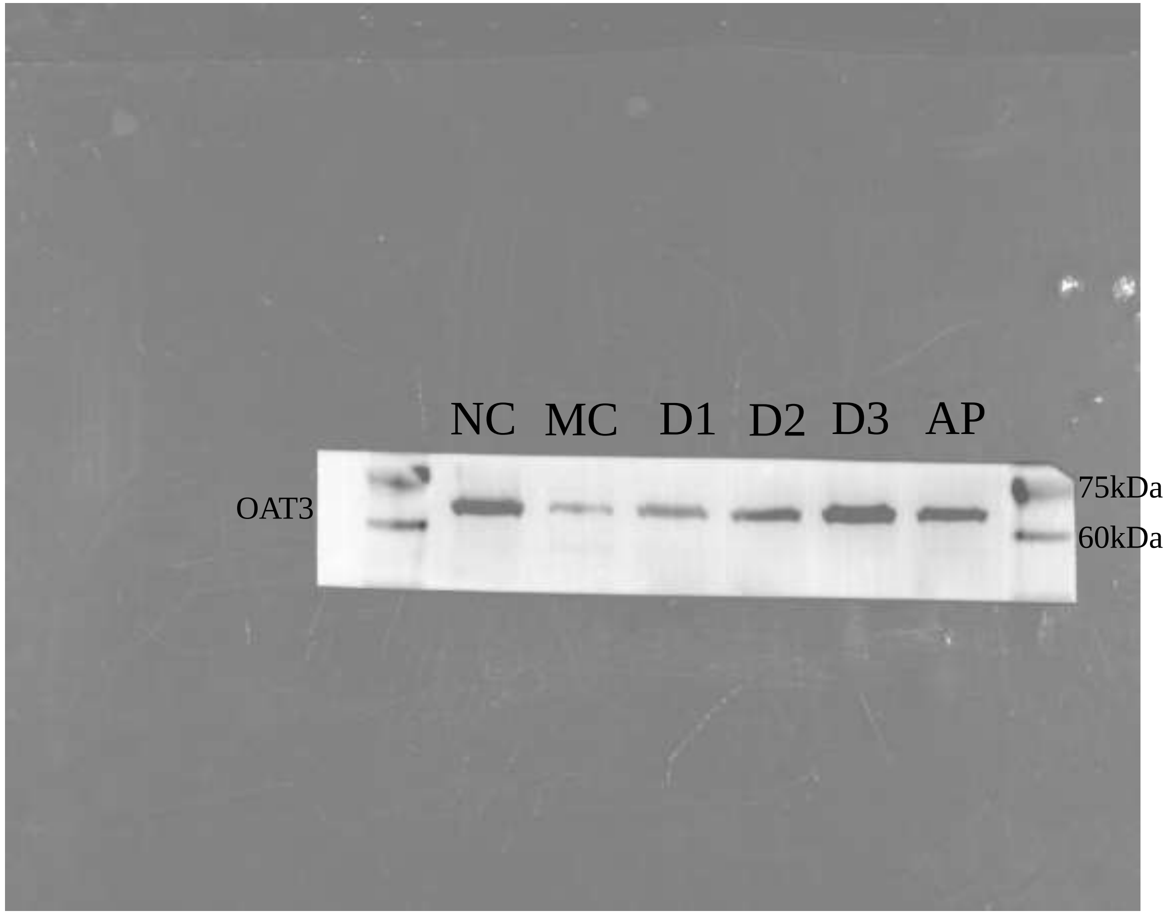

Supplement: Supplementary file 1 [file metabolites-14-00117-s001.zip › metabolites-2865971-supplementary.pdf]
